# Mobilization-based chemotherapy-free engraftment of gene-edited human hematopoietic stem cells

**DOI:** 10.1016/j.cell.2022.04.039

**Published:** 2022-06-23

**Authors:** Attya Omer-Javed, Gabriele Pedrazzani, Luisa Albano, Sherash Ghaus, Claire Latroche, Maura Manzi, Samuele Ferrari, Martina Fiumara, Aurelien Jacob, Valentina Vavassori, Alessandro Nonis, Daniele Canarutto, Luigi Naldini

**Affiliations:** 1 San Raffaele Telethon Institute for Gene Therapy, IRCCS San Raffaele Scientific Institute, Milan 20132, Italy; 2 Vita-Salute San Raffaele University, Milan 20132, Italy; 3 CUSSB—University Center for Statistics in the Biomedical Sciences, Vita-Salute San Raffaele University, Milan, Italy; 4 Pediatric Immunohematology Unit and BMT Program, IRCCS San Raffaele Scientific Institute, Milan, Italy

**Keywords:** CRISPR-Cas gene editing, hematopoietic stem cells, mobilization, X-linked hyper-IgM syndrome, human hematochimeric mouse model, RNA-based delivery, autologous stem cell transplantation, gene transfer, conditioning-free

## Abstract

Hematopoietic stem/progenitor cell gene therapy (HSPC-GT) is proving successful to treat several genetic diseases. HSPCs are mobilized, harvested, genetically corrected *ex vivo*, and infused, after the administration of toxic myeloablative conditioning to deplete the bone marrow (BM) for the modified cells. We show that mobilizers create an opportunity for seamless engraftment of exogenous cells, which effectively outcompete those mobilized, to repopulate the depleted BM. The competitive advantage results from the rescue during *ex vivo* culture of a detrimental impact of mobilization on HSPCs and can be further enhanced by the transient overexpression of engraftment effectors exploiting optimized mRNA-based delivery. We show the therapeutic efficacy in a mouse model of hyper IgM syndrome and further developed it in human hematochimeric mice, showing its applicability and versatility when coupled with gene transfer and editing strategies. Overall, our findings provide a potentially valuable strategy paving the way to broader and safer use of HSPC-GT.

## Highlights

• HSPC mobilizers create an opportunity to engraft exogenous cells in depleted niches
• *Ex vivo* culture endows HSPCs with migration advantage by rescuing CXCR4 expression
• Cultured HSPCs outcompete mobilized HSPCs for engraftment in depleted BM niches
• Transient engraftment enhancers coupled with gene editing confer a competitive advantage

## Graphical abstract

**Figure:**
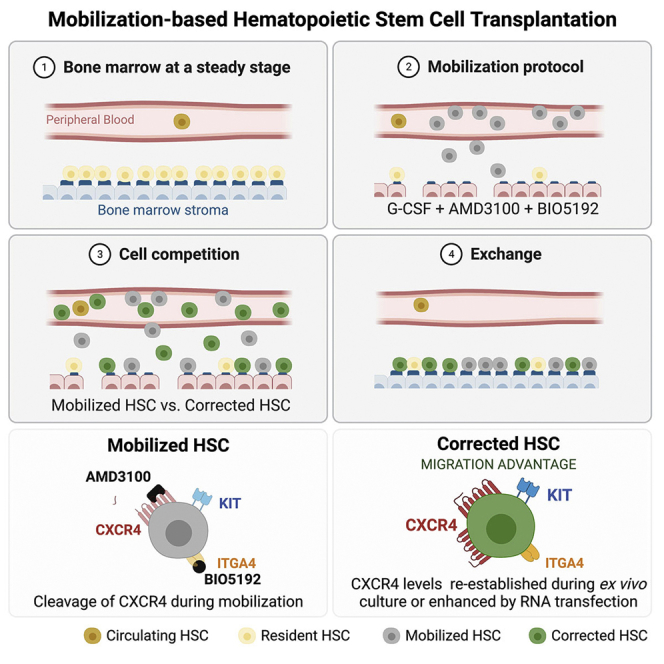

## Teaser

When transplanting engineered hematopoietic stem/progenitor cells, mobilizing the existing HSPCs out of the bone marrow can allow for the engraftment of new cells without the need to treat study animals with chemotherapy. This presents an opportunity to overcome the toxicity challenge that chemotherapy presents for HSPC transplantation in patients.

## Introduction

Hematopoietic stem cell transplantation (HSCT) is used to treat patients suffering from malignant and inherited diseases such as primary immunodeficiencies, Fanconi anemia, hemoglobinopathies, and lysosomal storage disorders (Copelan, 2006; Granot and Storb, 2020). However, its morbidity remains of concern, in particular, when considering its application to non-malignant diseases. The main cause of HSCT morbidity, when using an allogeneic source of hematopoietic stem/progenitor cells (HSPCs), resides in the life-threatening and debilitating graft versus host disease (GvHD) and in the long-term irreversible complications (such as secondary malignancies) arising from the genotoxic side effects of conditioning regimens, which are required to deplete the HSPCs residing in the bone marrow (BM) to make space for the donor HSPCs (Copelan et al., 2019). The development of effective gene correction methods promoted the use of autologous HSPCs to treat inherited diseases (Ferrari et al., 2021a). The best-established strategies for HSPC gene therapy (HSPC-GT) are based on gene replacement by integrating vectors, such as lentiviral vectors (LVs), which semi-randomly introduce one or more functional copies of the affected gene in the genomic DNA of targeted cells (Naldini, 2019). The recently emerged gene-editing tools enable site-specific deletions, insertions, nucleotide substitutions, and the targeted integration of a therapeutic transgene, allowing to suppress or, conversely, rescue the function and physiological expression of the targeted gene and offering the promise of more precise, versatile, and safer genetic manipulation (Doudna, 2020; Ferrari et al., 2021b).

Although autologous HSPC-GT eliminates the risk of GvHD, it maintains the requirement for partial or fully myeloablative conditioning. Current regimens involve non-specific, chemo-, or radio-therapeutic treatments that have multiple short- and long-term adverse effects and cause a prolonged immune suppression predisposing patients to severe and fatal infections (Gyurkocza and Sandmaier, 2014). These treatments also damage the BM stroma and HSPC niche architecture and may in turn adversely affect the extent and kinetics of cell engraftment. When the conditioning is milder, engraftment becomes a competitive process between endogenous and infused HSPCs (Andreani et al., 2011; Magnani et al., 2020; Socie et al., 1995; Walters et al., 2001). Several groups have thus investigated in mouse models the possibility of bypassing conditioning (Bhattacharya et al., 2006, 2009; Nilsson et al., 1997; Shimoto et al., 2017) by increasing the input of donor cells, also through *in vitro* expansion prior to infusion (Blomberg et al., 1998; Wilkinson et al., 2019). However, these strategies have proved to be either inefficient or are currently not compatible with the clinical use of human HSPCs. On the other hand, when endogenous HSPCs are depleted from the BM niche as in Fanconi’s anemia, HSCT and even HSPC-GT become possible without conditioning and show progressive hematopoietic repopulation by the infused wild-type (WT) or gene-corrected cells that bear a competitive advantage over the residual resident ones (Río et al., 2019).

A promising avenue for improving the safety of conditioning is the use of specific drugs that target HSPCs in the BM niche and spare non-hematopoietic cells. Several studies have focused on the use of monoclonal antibodies coupled or not with toxins and directed against cell surface antigens expressed by all white blood cells (WBCs), such as CD45 (Palchaudhuri et al., 2016; Schiroli et al., 2017), or HSPCs, such as KIT (Chhabra et al., 2016; Czechowicz et al., 2007; George et al., 2019; Kwon et al., 2019). Some of these strategies are now reaching clinical testing, which will enable a full assessment of their potential for application to different disease settings and transforming the current risk/benefit paradigm for HSCT (NCT02963064), although profound cytopenia might result from the degree of ablation needed for sufficient engraftment.

In adults, donor HSPCs, whether autologous or allogeneic, are harvested mainly by mobilizing regimes, which act by disrupting their interaction with the stroma in the BM niche (de Kruijf et al., 2020; Tay et al., 2017). The longest and most often used compound is granulocyte colony-stimulating factor (G-CSF), a cytokine regulating granulocytopoiesis and neutrophil function (Bendall and Bradstock, 2014). The administration of G-CSF acts at the level of BM in a broad and complex manner, which is not yet fully understood, and leads to a 6- to 7-fold increase of CD34^+^ cells in the circulation (Grigg et al., 1995). For mobilization purposes, G-CSF is administered once or twice daily for up to 6 days. Other molecules have been developed, such as AMD3100 (Plerixafor/Mozobil), a direct antagonist of CXCR4, which rapidly mobilizes HSPCs into the circulation by directly disrupting a major BM retention axis, CXCL12-CXCR4, in a reversible way (Aiuti et al., 1997; Winkler et al., 2012). The combination of G-CSF and AMD3100 synergistically increases CD34^+^ cells up to 10-fold above baseline levels in peripheral blood (PB) and is becoming a well-tolerated standard of care (Brave et al., 2010; Hsu and Cushing, 2016; Pusic and DiPersio, 2010). Other antagonists of HSPC surface adhesion receptors have also been explored (Domingues et al., 2017; Hoggatt and Pelus, 2012). ITGA4 antagonists, such as BIO5192, have been tested in combination with G-CSF and AMD3100, resulting in a 30-fold increase in mobilization of murine HSPCs over basal levels (Cao et al., 2016; Ramirez et al., 2009).

Ultimately, at least within the context of HSPC-GT, mobilization could also be exploited to lower the requirement for an extensive depletion of resident cells given that a stable partial chimerism with autologous gene-corrected cells is attainable and may be sufficient to provide therapeutic benefit in several non-malignant diseases (Zimmerman and Shenoy, 2020). Using parabiotic mice, it was shown that AMD3100 administration can promote the exchange of HSPCs between partners, albeit <5% (Chen et al., 2006). Two recent studies performed in mice exploited serial mobilization and transplantation cycles to enable the engraftment of donor HSPCs in the context of aging and Parkinson’s disease modeling (Chen et al., 2020; Guderyon et al., 2020). However, competition with residual cells in the recipient might be impaired when the infused cells underwent *ex vivo* genetic engineering. Culture conditions, exposure to viral vectors, and electroporation of editing machinery can variably induce HSPC differentiation or apoptosis and modify the expression of cell surface molecules relevant for BM homing and engraftment (Hall et al., 2006; Piras et al., 2017). In addition, DNA double-strand breaks (DSBs) induced by nuclease-based editors during HSPC gene editing, trigger a DNA damage response that limits hematopoietic repopulation and can be rescued in part transient inhibition of p53 (Ferrari et al., 2020; Schiroli et al., 2019).

Overall, despite substantial progress on the front of genetic engineering, the full therapeutic potential of HSPC-GT remains constrained by the requirement for toxic conditioning regimens. Here, we present a strategy leveraging efficient mobilization as a conditioning regimen, which allows engraftment of *ex vivo* gene-modified HSPCs to therapeutically meaningful levels without raising serious toxicity concerns. By taking advantage of an optimized mRNA delivery platform, human HSPCs are treated for gene transfer or gene editing and endowed with a transient engraftment advantage allowing stable long-term grafts in hematochimeric mouse models of human hematopoiesis to be established. These proof-of-principle studies should open the way to a potentially valuable approach to HSPC-GT.

## Results

### Long-term donor chimerism is established by mobilization-based HSCT

**Figure S1:**
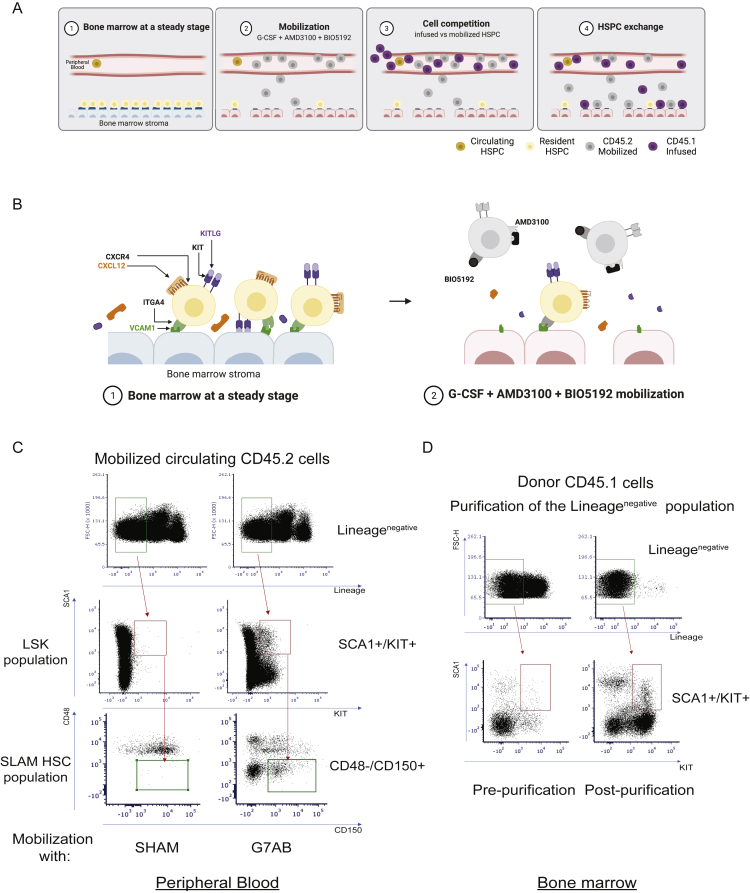
Long-term donor chimerism is established by M-HSCT, related to Figure 1 (A) Schematic of the M-HSCT strategy, illustrating the proposed exchange between mobilized recipient CD45.2 (in gray) and donor CD45.1 cells (in purple). (B) Schematic of the interaction between mobilization agents and their therapeutic targets within the BM microenvironment. (C) Representative plots showing the gating strategy used to characterize LSK and SLAM HSC circulating in the peripheral blood, stained for lineage markers, SCA1, KIT, CD48, and CD150. (D) Representative plots showing the gating strategy used to characterize the donor cells, extracted from the BM, stained for lineage markers, SCA1, KIT, pre-, and post-purification of lineage negative cells. In all the analyses, p less than 0.05 were considered significant (∗p < 0.05, ∗∗p < 0.01, ∗∗∗p < 0.001, ∗∗∗∗p < 0.0001. “ns” means non-significance).

**Figure 1:**
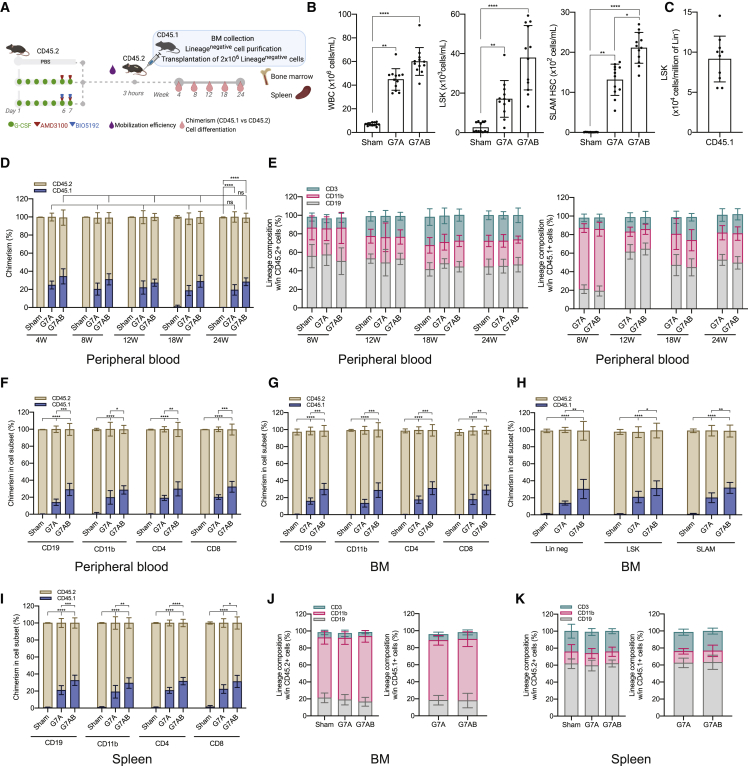
Long-term donor chimerism is established by mobilization-based HSCT (M-HSCT) (A) Schematic of the M-HSCT protocol. Recipient CD45.2 mice were mobilized with G-CSF (green dots) and AMD3100 (red triangle), with and without BIO5192 (blue triangle) (G7A; G7AB), and subsequently transplanted with CD45.1 2 × 10^6^ Lin^−^ cells, collected from the BM. (B) Counts of mobilized WBC (left panel), LSK (middle panel), and SLAM HSC (right panel) per mL in the PB in non-mobilized (Sham) and G7A and G7AB mobilized mice. (C) Counts of LSK cells per million of Lin^−^ CD45.1 donor cells, collected from the BM. (D) Long-term follow-up of the donor CD45.1 (blue) and recipient CD45.2 (ocher) chimerism observed within total CD45^+^ cells in PB after transplanting 2 × 10^6^ Lin^−^ cells post-mobilization in recipient CD45.2 mice. (E) The reconstitution of myeloid and lymphoid lineages over time of the recipient CD45.2^+^ cells (left panel) and CD45.1^+^ cells (right panel) in the PB of recipient CD45.2 mice. (F–I) Chimerism of CD45.1 cells was observed within CD19^+^ B cells, CD11b^+^ myeloid cells, CD4^+^ T helper cells, and CD8^+^ T cytotoxic cells in the PB (F) and BM (G), within the Lin^−^, LSK, and SLAM HSC (H) and spleen (I). (J and K) Myeloid and lymphoid lineage composition of CD45.2^+^ cells (left panel) and CD45.1^+^ cells (right panel) in the BM (J) and spleen (K) of CD45.2 mice. The results are mean ± SEM, with n ≧ 10 biological replicates. In all the analyses, p less than 0.05 were considered significant (∗p < 0.05, ∗∗p < 0.01, ∗∗∗p < 0.001, ∗∗∗∗p < 0.0001. “ns” means non-significance).

We hypothesized that mobilization regimens, while inducing substantial egress of resident HSPCs from the BM, might also, per se, avail space for newly transplanted cells. Thus, transplanting HSPCs at the peak of mobilization might enable competition with mobilized recipient cells to repopulate the BM niches, establishing some levels of donor chimerism (Figure S1A). We tested two mobilization regimens in mice, one modeling a clinically approved protocol using G-CSF and AMD3100 (G7A) and the other one also comprising BIO5192 (G7AB). C57BL/6J CD45.2 mice were treated with G-CSF using a subcutaneous pump for a week combined with either AMD3100 or the combination of AMD3100 and BIO5192 delivered intraperitoneally on days 6 and 7 (Figure 1A; Figure S1B). We assessed mobilization 3 h post-AMD3100 injection and showed a 6-fold increase in WBC counts, an 8-fold increase in progenitors (Lin^−^SCA1^+^KIT^+^ [LSK]), and a 200-fold increase in the HSC-enriched fraction (LSK CD150^+^CD48^−^ [SLAM HSC]) in the circulation (Figure 1B; Figure S1C), compared with non-mobilized control mice (Sham). As reported in the literature (Ramirez et al., 2009), the addition of BIO5192 led to a higher mobilization of WBC, LSK, and SLAM HSC, leading to an additional 2-fold increase of SLAM HSC over G7A levels.

To test if these levels of mobilization, and the subsequent BM niche availability, were sufficient to allow engraftment of donor cells, 2 × 10^6^ CD45.1 lineage negative (Lin^−^) cells, purified from the BM, were transplanted into CD45.2 congenic recipients after the last injection of AMD3100 or AMD3100/BIO5192 (Figures 1A and 1C; Figure S1D). Donor chimerism reached a median of 20% following the G-CSF/AMD3100 mobilization protocol, and the addition of BIO5192 further increased it to 30%. Chimerism was stable for up to 24 weeks (Figure 1D), while only a minimal donor chimerism was observed in non-mobilized control mice (<1%). The myeloid and lymphoid proportion was similar between mobilized and non-mobilized control mice (Figure 1E, left panel). Donor cells showed a myeloid skewed output in the first weeks after engraftment, likely consistent with early repopulation by short-term progenitors (Figure 1E, right panel). However, by the end of the experiment, donor cells and recipient cells were similar in terms of lineage composition (Figure 1E). The chimerism was maintained among differentiated cell subsets, i.e., T cells (CD4 and CD8), B cells (CD19), and myeloid cells (CD11b) in the PB (Figure 1F), BM (Figure 1G), including within LSK and SLAM HSC (Figure 1H), and spleen (Figure 1I), and the lineage distribution was similar for cells of recipient and donor origin in both organs (Figures 1J and 1K). Overall, these data support the engraftment of donor long-term multilineage HSPCs. These findings established the proof of principle for a low-burden HSCT, which entails an exchange of residents with donor HSPCs during mobilization without genotoxic conditioning, hereafter referred to as mobilization-enabled HSCT (M-HSCT).

### M-HSCT allows establishing sufficient donor chimerism to rescue the HIGM-1 phenotype

**Figure 2:**
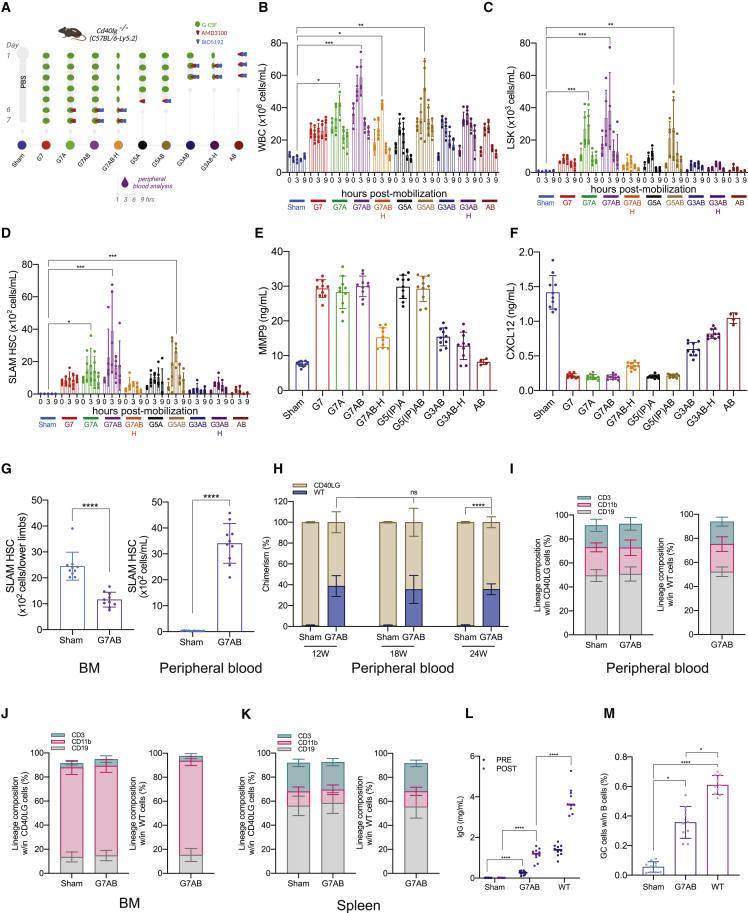
M-HSCT allows establishing sufficient donor chimerism to rescue the HIGM1 phenotype (A) Schematic of different mobilization protocols tested and the times of analysis in *Cd40lg*^*−/−*^ mice. (B–D) Counts of mobilized WBC (B), LSK (C), and SLAM HSC (D) per mL in the PB of *Cd40lg*^*−/−*^ mice treated with PBS (Sham), G-CSF for 7 days (G7), G-CSF for 7 days and AMD3100 (G7A), G-CSF for 7 days, AMD3100 and BIO5192 (G7AB), half-dose of G-CSF for 7 days, AMD3100 and BIO5192 (G7AB-H), G-CSF for 5 days and AMD3100 (G5A), G-CSF for 5 days, AMD3100 and BIO5192 (G5AB), G-CSF for 3 days, AMD3100 and BIO5192 (G3AB), half-dose of G-CSF for 3 days, AMD3100 and BIO5192 (G3AB-H), and only AMD3100 and BIO5192 (AB) at 0, 1, 3, 6, and 9 h after the last injection of A or AB. (E and F) MMP9 (E) and CXCL12 (F) concentration in the BM extracellular extracts of *Cd40lg*^*−/−*^ mice mobilized with protocols described above. (G) Total counts of SLAM HSC in the lower limbs (left panel) and in the PB per mL (right panel) of *Cd40lg*^*−/−*^ mice treated with PBS or mobilized with G7AB. (H) Long-term follow-up of the donor WT (blue) and recipient *Cd40lg*^*−/−*^ (ocher) chimerism observed within total CD45^+^ cells in PB after transplanting 2 × 10^6^ Lin^−^ cells (collected from the BM) post mobilization in recipient *Cd40lg*^*−/−*^ mice. (I–K) Myeloid and lymphoid lineage composition of *Cd40lg*^*−/−*^ (left panel) and WT (right panel) cells in the PB (I), BM (J), and spleen (K) of *Cd40lg*^*−/−*^ mice after M-HSCT. (L) TNP-KLH-specific IgG concentration in sera collected 7 days before (pre) and after (post) TNP-KLH vaccination of *Cd40lg*^*−/−*^ mice after M-HSCT. (M) The percentage of PNA^+^GL7^+^ splenic germinal centers B cells within the spleen of *Cd40lg*^*−/−*^ mice after TNP-KLH vaccination of *Cd40lg*^*−/−*^ mice treated by M-HSCT. The results are mean ± SEM, with n ≧ 5 biological replicates for the kinetic experiments, except for the AB group (n = 4), and with n ≧ 9 biological replicates for the *Cd40lg*^*−/−*^ M-HSCT experiments. In all the analyses, p less than 0.05 were considered significant (∗p < 0.05, ∗∗p < 0.01, ∗∗∗p < 0.001, ∗∗∗∗p < 0.0001. “ns” means non-significance).

**Figure S2:**
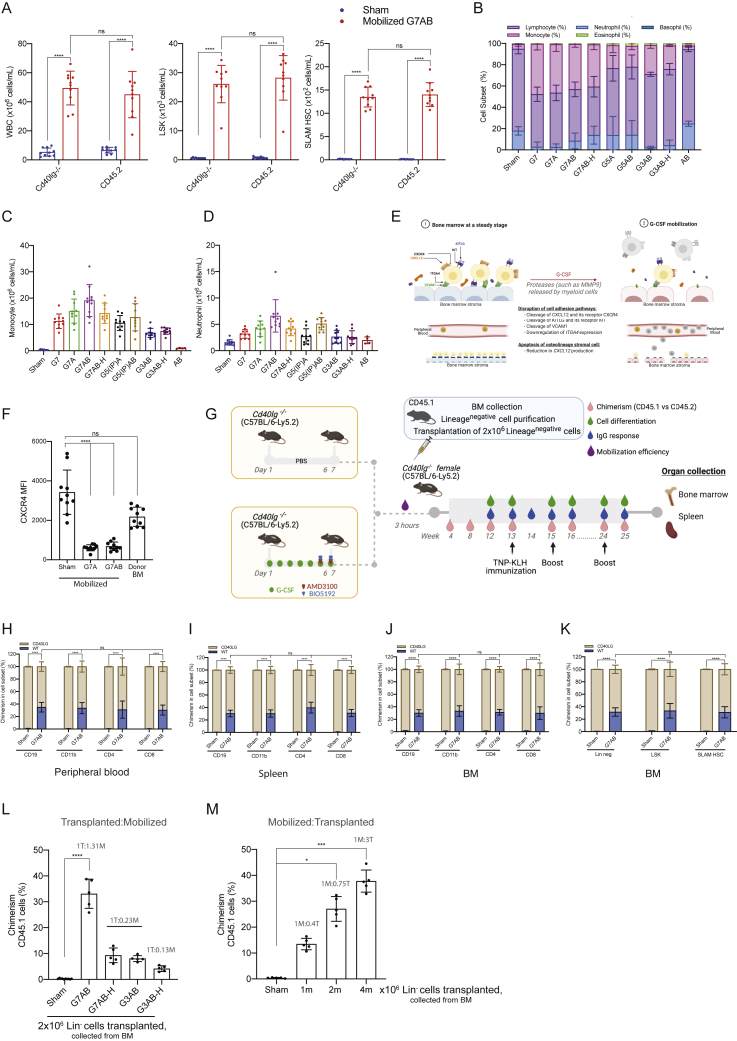
M-HSCT allows establishing sufficient donor chimerism to rescue the HIGM1 phenotype, related to Figure 2 (A) Counts of mobilized WBC (left panel), LSK (middle panel), and SLAM HSC (right panel) cells per mL in the PB of *Cd40lg*^*−/−*^ mice and CD45.2 mice, after mobilization with PBS (Sham) or mobilized with G7AB. A mixed-effect model (REML), followed by a post hoc analysis with Sidak’s test. (B) The percentage of neutrophils, lymphocytes, monocytes, eosinophils, and basophils in the PB after different mobilization protocols. (C and D) Counts of mobilized monocytes (C) and neutrophils (D) per mL in the PB after different mobilization protocols. (E) Schematic of G-CSF impact on the BM. (F) CXCR4 MFI on circulating LSK after treatments with PBS, G7A, G7AB, and on donor cells purified from untreated bone marrow. A Kruskal-Wallis test, followed by a post hoc analysis with Dunn’s test. (G) Schematic of the M-HSCT protocol applied to *Cd40lg*^*−/−*^ mice. The recipient *Cd40lg*^*−/−*^ mice were mobilized with G-CSF (green dots), AMD3100 (red triangle), and BIO5192 (blue triangle) (G7AB), and subsequently transplanted with 2 × 10^6^ WT CD45.1 Lin^−^ cells, collected from the BM. (H–K) Chimerism of WT cells was observed within CD19^+^, CD11b^+^, CD4^+^, and CD8^+^ cells in the PB (H), spleen (I), and BM (J) and within Lin^−^, LSK, and SLAM HSC in the BM (K). A mixed-effects model (REML), followed by a post hoc analysis with Tukey’s test (between groups) or by post hoc analysis with Dunnett’s test (within groups). (L) Chimerism of CD45.1 cells at 20 weeks, following different M-HSCT protocol, subsequently transplanted with 2 × 10^6^ WT CD45.1 Lin^−^ cells, collected from the BM. (M) Chimerism of CD45.1 cells at 20 weeks, following mobilization with G7AB and subsequently transplanted with different cell doses (WT CD45.1 Lin^−^ cells, collected from the BM). Results are mean ± SEM, with n ≧ 5 for the kinetic experiments, except for the AB group (n − 4), and with n ≧ 9 for the *Cd40lg*^−*/*−^ M-HSCT experiments. In all the analyses, p less than 0.05 were considered significant (∗p < 0.05, ∗∗p < 0.01, ∗∗∗p < 0.001, ∗∗∗∗p < 0.0001. “ns” means non-significance).

To investigate the therapeutic potential of M-HSCT, we took advantage of *Cd40lg*^*−/−*^ mice, which faithfully recapitulate the phenotype of the human primary combined immunodeficiency hyper IgM syndrome 1 (HIGM-1; Renshaw et al., 1994). Because G-CSF treatment may have a detrimental effect on the BM niche (Greenbaum and Link, 2011; Lévesque et al., 2003a, 2003b; Petit et al., 2002), we investigated the efficiency of mobilization protocols differing in its presence or absence, the G-CSF dose and the duration, and combination with other drugs (Figure 2A). Removing the G-CSF or reducing its dose or treatment time significantly decreased the mobilization of LSK and SLAM HSC. PB analysis revealed a peak of mobilization of WBC, LSK, and SLAM HSC around 3 h post-AMD3100 or AMD3100/BIO5192 injection (Figures 2B–2D). The addition of AMD3100 to the standard G-CSF treatment increased the mobilized LSK by 3-fold, and BIO5192 further increased it by 5-fold (Figure 2C). The levels of mobilization observed in *Cd40lg*^*−/−*^ mice were comparable to those obtained in the WT mice (Figure S2A). Although the administration of only AMD3100/BIO5192 had a limited impact on blood neutrophils and monocytes counts, they were highly increased after the G-CSF treatment (Figures S2B–S2D). Moreover, the G-CSF treatment increased MMP9, a protease released by myeloid cells, and decreased CXCL12, a chemokine involved in HSPC homing and retention, in the BM stroma, confirming the remodeling of the extracellular matrix (Figures 2E and 2F; Figure S2E), as reported in the literature (Greenbaum and Link, 2011). Intriguingly, when we compared the level of CXCR4 expression on circulating SLAM HSC with or without G-CSF mobilization, we found that it was lower in the former condition, suggesting the induced cleavage of the molecule (Figure S2F). By comparing the number of SLAM HSC in lower limb BM of untreated and mobilized mice, at the peak of mobilization, we found a 55% decrease in the latter condition (Figure 2G; left panel). However, only 75% of these cells could be accounted for in the PB (Figure 2G; right panel), suggesting that some of the egressed cells became trapped in other organs, such as the spleen.

Since current editing protocols are considerably less efficient and more detrimental for mouse cells than for their human counterpart (Schiroli et al., 2017), we transplanted 2 × 10^6^ WT CD45.1 Lin^−^ cells, purified from the BM, as a surrogate of autologous gene-corrected cells and transplanted them into *Cd40lg*^*−/−*^ recipients at the peak of mobilization following the G-CSF, AMD3100, and BIO5192 regimen (Figure S2G). A stable chimerism of about 30% WT cells was reached across all lineages and differentiated cells in the PB (Figure 2H; Figure S2H), spleen (Figure S2I), and BM (Figures S2J and S2K) of transplanted mice. By the end of the experiment, cellular subsets were comparable in recipient versus donor cells and between the non-mobilized (Sham) and mobilized group (G7AB) in the PB (Figure 2I), BM (Figure 2J), and spleen (Figure 2K).

Intriguingly, the chimerism experimentally obtained was higher than our estimate of approximately 20% based on data from Figure 2G and the following postulates: (1) mobilization occurred to the same extent throughout all the mouse BM, (2) the lower limbs account for 20% of it (Nombela-Arrieta and Manz, 2017), and (3) the mobilized and infused cells compete equally (Table 1; column G7AB). This finding suggests an advantage of the cells harvested from the donor BM and not exposed to the mobilization procedure, possibly conferred by the higher expression of CXCR4 (Figure S2F) in the context of a limiting amount of CXCL12, highlighting a favorable window of opportunity for engraftment of donor HSPCs right upon mobilization.

**Table 1:** Estimated versus obtained chimerism following M-HSCT in *Cd40lg*^*−/−*^ mice

Treatment	Mobilized SLAM HSCs (mL)	Estimated mobilized SLAM HSCs/mouse (V blood = 1.5 mL)	Percentage of estimated BM vacancy	Total SLAM HSCs transplanted (from BM purified 2 × 10^6^ Lin^−^)	Ratio recipient to donor	Percentage of estimated chimerism	Percentage of obtained chimerism
Sham	10	15	0	4,000	1:0.00375	0	N/A
G7	900	1,350	11	4,000	1:0.33	8	N/A
G7A	1,700	2,550	20	4,000	1:0.64	12	19
G7AB	3,500	5,250	42	4,000	1:1.31	18	30
G7AB-H	600	900	7	4,000	1:0.23	6	9
G5(IP)A	1,000	1,500	12	4,000	1:0.38	9	N/A
G5(IP)AB	2,500	3,750	30	4,000	1:0.94	15	N/A
G3AB	600	900	7	4,000	1:0.23	6	8
G3AB-H	350	525	4	4,000	1:0.13	4	5
AB	300	450	4	4,000	1:0.11	3	N/A

Estimation is based on the following theories: (1) mobilization occurred to the same extent throughout all the mouse BM, (2) the lower limbs account for 20% of it, and (3) the mobilized and infused cells compete equally (with n ≧ 5).

To investigate the dynamic of niche repopulation in the context of mobilization, we transplanted 2 × 10^6^ WT CD45.1 Lin^−^ cells, purified from the BM, following different mobilization protocols. We observed that despite transplanting the same number of cells, the chimerism falls with lower mobilization efficiency. Despite infusing 7 times more donor cells than mobilized ones, the chimerism remained low, indicating that residual non-mobilized cells are limiting the exchange, most likely by occupying the niche (Table 1; Figure S2L). On the other hand, when varying the numbers of transplanted cells below the saturating dose in mice mobilized with the same protocol, we found dose-dependent engraftment (Figure S2M).

We then vaccinated the mice with a thymus-dependent antigen (trinitrophenyl-keyhole limpet hemocyanin, TNP-KLH) and measured whether immunoglobulin (IgG) class switching was restored by the transplantation. Whereas non-mobilized *Cd40lg*^*−/−*^ mice produced nearly undetectable amounts of antigen-specific IgGs, M-HSCT-treated mice showed a significant rescue in switched-antibody responses to the primary and recall vaccinations, albeit reaching levels below those of control WT mice (Figure 2L). Partial rescue of immune function in transplanted mice was also shown by the presence of splenic germinal B cells, whereas non-mobilized mice failed to engage B cells for germinal center formation (Figure 2M).

Overall, these findings show the potential of M-HSCT for the rescue of the humoral immune response in HIGM-1 mice without the requirement of a conditioning regimen.

### M-HSCT allows an efficient donor to recipient exchange of HSPCs within the human niche of hematochimeric mice

**Figure 3:**
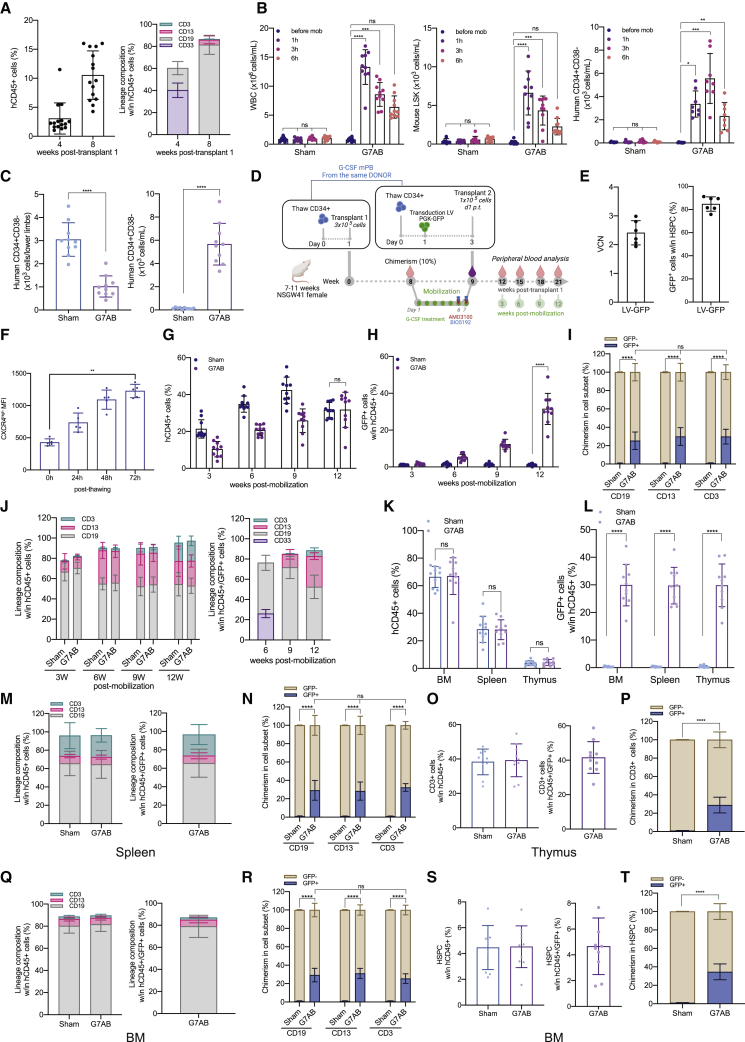
M-HSCT allows an efficient donor to recipient exchange of HSPCs within the human niche of hematochimeric mice (A) The percentage of human chimerism (CD45^+^; left panel) and lymphoid/myeloid cell composition within the human CD45^+^ population (right panel) over time in NSGW41 mice, following the first transplant of human CD34^+^ cells. (B) Counts of mobilized WBC (left panel), LSK cells (middle panel), and human CD34^+^ CD38^−^ cells (right panel) per mL in the PB of humanized NSGW41 mice non-mobilized or mobilized with G7AB at 0, 1, 3, and 6 h after the last injection of AMD3100 and BIO5192. (C) Total counts of CD34^+^ CD38^−^ cells in the lower limbs (left panel) and the PB per mL (right panel) of humanized NSGW41 mice non-mobilized (Sham) or mobilized (G7AB). (D) Schematic illustration of competitive transplantation, after G7AB mobilization, between human resident cells and newly transplanted CD34^+^ G-CSF mPB GFP-transduced cells in NSGW41 mice. (E) Vector copy number (VCN) in HSPCs population (CD34^+^CD133^+^CD90^+^) (left panel) and the percentage of GFP^+^ cells measured within CD34^+^ cells (right panel), *in vitro*, after transduction. (F) CXCR4^high^ MFI over time after thawing, stained with an antibody targeting the N terminus epitope of CXCR4, on the HSPC population (CD34^+^CD133^+^CD90^+^) mobilized with G-CSF. (G and H) Long-term follow-up of human CD45^+^ (G) and GFP^+^/CD45^+^(H) cells chimerism in PB after M-HSCT in NSGW41 mice. (I) Chimerism of GFP^+^ cells was observed within CD19^+^, CD13^+^, and CD3^+^ cells at the end of the experiment in PB after M-HSCT. (J) The reconstitution of myeloid and lymphoid lineages over time of human CD45^+^ (left panel) and CD45^+^/GFP^+^ cells (right panel) in the PB after M-HSCT. (K and L) Human CD45^+^ (K) and GFP^+^/CD45^+^ (L) cells chimerism in the BM, spleen, and thymus after M-HSCT. (M) Myeloid and lymphoid lineage composition of human CD45^+^ cells (left panel) and CD45^+^/GFP^+^ cells (right panel) in the spleen of NSGW41 mice after M-HSCT. (N) Chimerism of GFP^+^ cells was observed within CD19^+^, CD13^+^, and CD3^+^ cells in the spleen after M-HSCT. (O) The percentage of T cells within human CD45^+^ (left panel) and CD45^+^/GFP^+^ cells (right panel) in the thymus after M-HSCT. (P) Chimerism of GFP^+^ cells was observed within T cells in the thymus after M-HSCT. (Q) Myeloid and lymphoid lineage composition of human CD45^+^ (left panel) and CD45^+^/GFP^+^ cells (right panel) in the BM of NSGW41 mice after M-HSCT. (R) Chimerism of GFP^+^ cells was observed within CD19^+^, CD13^+^, and CD3^+^ cells in the BM after M-HSCT. (S) The percentage of HSPCs (CD34^+^ CD38^−^ CD90^+^) in human CD45^+^ (left panel) and CD45^+^/GFP^+^ cells (right panel) in the BM after M-HSCT. (T) Chimerism of GFP^+^ cells was observed within HSPCs in the BM after M-HSCT. The results are mean ± SEM, with n ≧ 9 biological replicates. In all the analyses, p less than 0.05 were considered significant (∗p < 0.05, ∗∗p < 0.01, ∗∗∗p < 0.001, ∗∗∗∗p < 0.0001. “ns” means non-significance).

**Figure S3:**
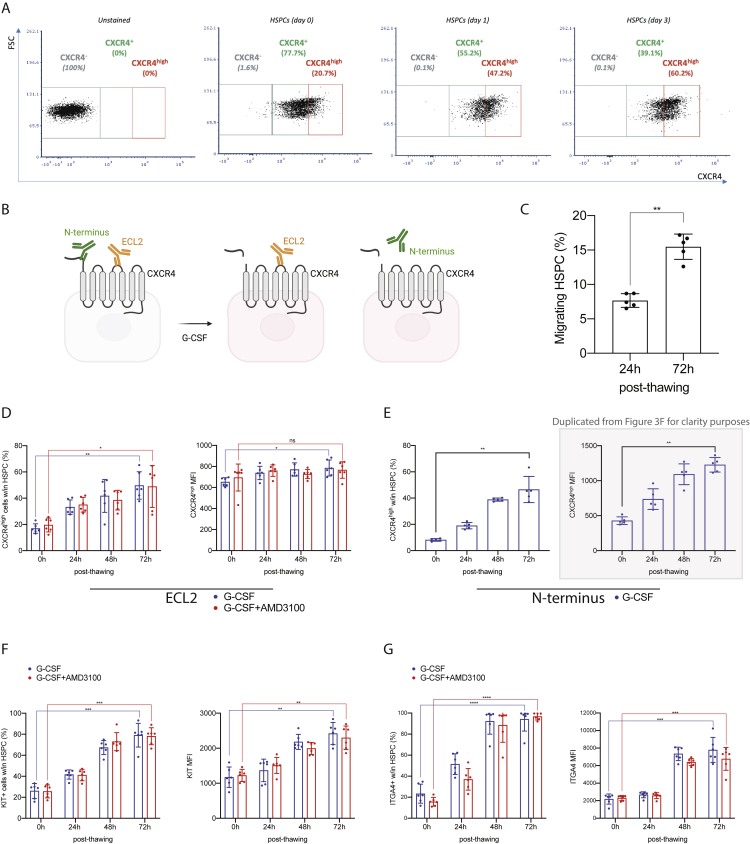
M-HSCT allows an efficient donor to recipient exchange of HSPCs within the human niche of hematochimeric mice, related to Figure 3 (A) Representative plots showing the gating strategy used to characterize the CXCR4^high^ population in the HSPC (CD34^+^CD133^+^CD90^+^) population. (B) Scheme of CXCR4 cleavage following G-CSF mobilization and related antibodies localization. (C) The percentage of migrating HSPCs, performed at 24 and 72 h post thawing, previously collected with G-CSF. A Kruskal-Wallis test was performed, followed by a post hoc analysis with Dunn’s test. (D) The percentage of CXCR4^high^ cells (left panel) and MFI (right panel) over time after thawing, stained with an antibody targeting the ECL2 epitope of CXCR4, on the HSPC population mobilized with G-CSF or G-CSF/AMD3100. (E) The percentage of CXCR4^high^ cells (left panel) and MFI (right panel) over time after thawing, stained with an antibody targeting the N terminus epitope of CXCR4, on the HSPC population mobilized with G-CSF. (F) The percentage of KIT^+^ cells (left panel) and MFI (right panel) over time after thawing, on the HSPC population mobilized with G-CSF or G-CSF/AMD3100. (G) The percentage of ITGA4^+^ cells (left panel) and MFI (right panel) over time after thawing, on the HSPC population mobilized with G-CSF or G-CSF/AMD3100. The longitudinal comparisons, performed by a mixed-effect model (REML), followed by a post hoc analysis with Dunnett’s test. The results are mean ± SEM, with n ≧ 5, with 3 different donors. In all the analyses, p less than 0.05 were considered significant (∗p < 0.05, ∗∗p < 0.01, ∗∗∗p < 0.001, ∗∗∗∗p < 0.0001. “ns” means non-significance).

Next, we embarked on modeling the feasibility of M-HSCT in humans by using hematochimeric NOD.Cg-Kit^W41J^ Prkdc^scid^ Il2rg^tm1Wjl^/WaskJ (NSGW41) mice, which harbor a mutant kit receptor decreasing the competition with human HSPCs. We first set up an *in vivo* model of human HSPC mobilization. NSGW41 mice were transplanted with 3 × 10^5^ G-CSF mobilized PB (mPB) derived CD34^+^ cells and, once hematopoietic engraftment was established (Figure 3A), mice were treated with the combination mobilization protocol optimized above (G7AB). Mobilization led to a substantial increase in WBC counts, murine (LSK), and human (CD34^+^CD38^−^) progenitors (Figure 3B). Of note, the mobilized human CD34^+^CD38^−^ cells peaked 3 h after the last administration of mobilizers, suggesting this time as the best candidate for transplanting human donor cells. In line with PB results, we showed up to a 65% decrease in human CD34^+^CD38^−^ cells in the BM of mobilized mice. This decrease was concurrent with an increase of human CD34^+^CD38^−^ cells in PB, accounting for 60% of the egressed cells (Figure 3C). We then tested CD34^+^CD38^−^ cell exchange post mobilization, by transplanting the outgrowth of 1 × 10^5^ CD34^+^ cells, counted on day 1 post thawing (Figure 3D). Prior to infusion, the mPB cells from the same donor were transduced with GFP-expressing LV (Figures 3D–3E), to distinguish them from the previously engrafted and now resident cells. This culture step, which models an LV-based gene replacement protocol for therapeutic purposes, led to an increased expression of CXCR4, KIT, and ITGA4 (all involved in HSPC homing and retention), counteracting the effect of thawing and prior *in vivo* G-CSF exposure (Figure 3F; Figures S3A–S3G). Intriguingly, the mobilized cells showed increasing CXCR4 MFI upon culture when immunostaining with an N terminus-directed antibody, consistent with the reported N-terminal cleavage of the molecule upon exposure to G-CSF and rescued expression of the full-size molecule in culture (Lévesque et al., 2003a, 2003b). The size of the human graft continued to increase over time in non-mobilized mice but comprised only a low % of GFP^+^ cells (<1%) even at late time points, showing that in the absence of mobilization, there is no engraftment of the newly infused cells (Figure 3G). On the contrary, when mice received the mobilization treatment, the human graft initially decreased but was then followed by a rescue accompanied by an increasing fraction of GFP^+^ cells, suggesting that these cells were able to effectively compete with the mobilized ones. At the end of the experiment, the GFP^+^ cells were a median of 30% of the human graft and found within all human lineages present in the blood of mobilized mice (CD19^+^ B cells, CD13^+^ myeloid cells, and CD3^+^ T cells) while being nearly absent in non-mobilized ones (Figures 3G–3I). Lineage composition was similar between mobilized and non-mobilized mice (Figure 3J, left panel). Interestingly, the GFP^+^ cells showed a myeloid skewed output in the first weeks after transplantation, as observed for newly engrafting progenitors in the C57BL/6J mouse model above (Figure 3J, right panel). However, GFP^+^ cells were found in all detectable lineages in the following weeks and approached the lineage distribution of the total human graft at the end of the experiment (Figure 3J). In the BM, spleen, and thymus, the human graft levels were similar in mobilized and non-mobilized mice, whereas the GFP^+^ cells reached up to 30% in the mobilized mice (Figures 3K–3L). The non-mobilized mice had GFP^+^ engraftment below 1%. The lineage distribution between the total human graft and the GFP^+^ cells was similar in all the organs (Figures 3M–3R), with comparable levels of chimerism across all cell subsets, including the most primitive progenitors (HSPCs: CD34^+^CD38^−^CD90^+^; Figures 3S and 3T), indicating stable and functional engraftment. These data support the hypothesis that partial chimerism of *ex vivo* modified HSPCs can be established for human hematopoiesis following M-HSCT and without prior conditioning.

### Transient overexpression of CXCR4 increases chimerism in the humanized context, following M-HSCT

**Figure S4:**
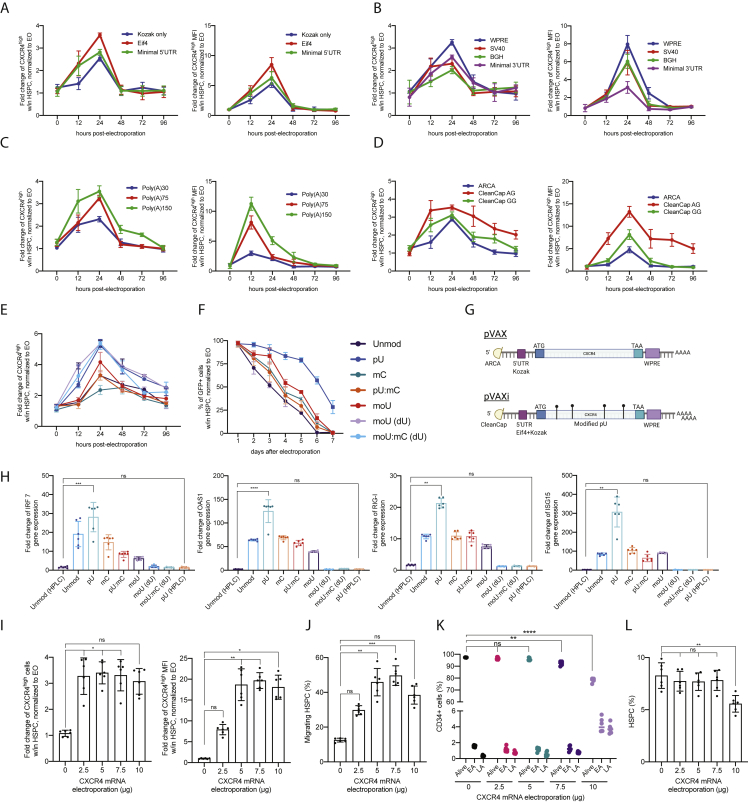
Optimization of the mRNA delivery platform, related to Figure 4 (A–D) Fold change of CXCR4^high^ cells (left panel) and CXCR4^high^ MFI (right panel) in HSPCs (CD34^+^CD133^+^CD90^+^) electroporated with *CXCR4* mRNA differing for the 5′ UTR sequence (A), the 3′ UTR sequence (B), the polyA tail length (C), and the mRNA capping (D), normalized to electroporated-only (EO) cells. (E) Fold change of CXCR4^high^ cells in HSPCs electroporated with *CXCR4* mRNA differing for nucleotides used during mRNA synthesis, normalized to EO cells. (F) The percentage of GFP^+^ cells in HSPCs, electroporated with *GFP* mRNA differing for nucleotides used for the mRNA synthesis, normalized to EO cells. (G) Schematic of the pVAX CXCR4 mRNA and improved pVAXi CXCR4 mRNA. (H) Fold change of IRF7, OAS1, RIG-I, and ISG15 gene expression, in cells electroporated with *CXCR4* mRNA differing for nucleotides used during mRNA synthesis, normalized to EO cells. (I) Fold change of CXCR4^high^ cells (left panel) and CXCR4^high^ MFI (right panel) in HSPCs, electroporated with different quantities of *CXCR4* mRNA, normalized to EO cells. (J) The percentage of migrating HSPCs, electroporated with different quantities of *CXCR4* mRNA. (K) Early and late apoptosis (EA and LA) is induced by the electroporation of different quantities of *CXCR4* mRNA, in bulk CD34^+^ cells. (L) The percentage of HSPCs (CD34^+^CD133^+^CD90^+^) in CD34^+^ cells electroporated with different quantities of *CXCR4* mRNA. A Kruskal-Wallis test, followed by a post hoc analysis with Dunn’s test (G and K). The results are mean ± SEM, with n ≧ 5 for data collected *in vitro* (3 different donors). In all the analyses, p less than 0.05 were considered significant (∗p < 0.05, ∗∗p < 0.01, ∗∗∗p < 0.001, ∗∗∗∗p < 0.0001. “ns” means non-significance).

**Figure 4:**
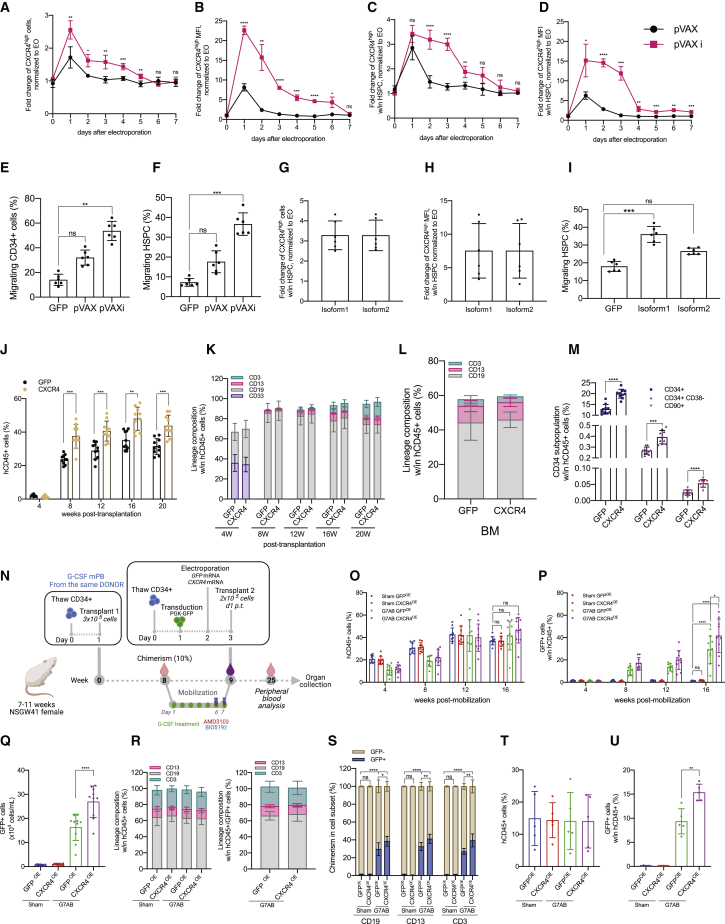
The transient overexpression of CXCR4 increases chimerism in the humanized context, following M-HSCT (A and B) Fold change of CXCR4^high^ cells (A) and CXCR4^high^ MFI (B) in bulk CD34^+^ cells, electroporated with *CXCR4* mRNA (pVAX) or optimized *CXCR4* mRNA (pVAXi), normalized to electroporated-only (EO) cells. (C and D) Fold change of CXCR4^high^ cells (C) and CXCR4^high^ MFI (D) in CD34^+^CD133^+^CD90^+^ HSPCs, electroporated with *CXCR4* mRNA or optimized *CXCR4* mRNA, normalized to EO cells. (E and F) The percentage of migrating bulk CD34^+^ cells (E) and HSPCs (F), electroporated with *GFP*, *CXCR4*, or optimized *CXCR4* mRNA. (G and H) Fold change of CXCR4^high^ cells (G) and CXCR4^high^ MFI (H) in HSPCs, electroporated with *CXCR4* isoform1 or isoform2 mRNA, normalized to EO cells. (I) The percentage of migrating HSPCs, electroporated with *GFP*, *CXCR4* isoform1, or isoform2 mRNA. (J) Long-term follow-up of human CD45^+^ in the PB of NSG mice, following the transplantation of CD34^+^ cells electroporated with *GFP* or *CXCR4* mRNA. (K) The reconstitution of myeloid and lymphoid lineages over time within total CD45^+^ cells in the PB of NSG mice, transplanted with CD34^+^ cells electroporated with *GFP* or *CXCR4* mRNA. (L) Myeloid and lymphoid cell composition of human CD45^+^ cells in the BM of NSG mice, transplanted with CD34^+^ cells electroporated with *GFP* or *CXCR4* mRNA. (M) CD34^+^ subpopulation in the BM of NSG mice, transplanted with CD34^+^ cells electroporated with *GFP* or *CXCR4* mRNA. (N) Schematic illustration of competitive transplantation, after G7AB mobilization, between human resident cells and newly transplanted CD34^+^ G-CSF mPB GFP-transduced and electroporated cells in NSGW41 mice. (O and P) Long-term follow-up of human CD45^+^ (O) and CD45^+^/GFP^+^ (P) cells in the PB after M-HSCT with CD34^+^ cells transiently overexpressing *GFP* or *CXCR4* mRNA, in NSGW41 mice. (Q) Counts of GFP^+^ cells per mL in the PB after M-HSCT with CD34^+^ cells transiently overexpressing *GFP* or *CXCR4* mRNA, in NSGW41 mice. (R) Myeloid and lymphoid lineage composition of human CD45^+^ (left panel) and CD45^+^/GFP^+^ cells (right panel) in the PB after M-HSCT, with CD34^+^ cells transiently overexpressing GFP or CXCR4, in NSGW41 mice. (S) Chimerism of GFP^+^ cells was observed within CD19^+^, CD13^+^, and CD3^+^ cells in the PB after M-HSCT, with CD34^+^ cells transiently overexpressing GFP or CXCR4, in NSGW41 mice. (T) The percentage of human CD45^+^ cells in the PB of NSG mice at 16 weeks, following the secondary transplant of cells collected from groups described in Figure 4O. (U) Percentage of human CD45^+^/GFP^+^ cells in PB of NSG mice at 16 weeks, following the secondary transplant of cells collected from groups described in Figure 4O. The results are mean ± SEM, with n ≧ 10 biological replicates for data collected *in vivo*, n ≧ 4 biological replicates for secondary transplant experiments, and n ≧ 5 for data collected *in vitro* (3 biological replicates and 2 technical replicates). In all the analyses, p less than 0.05 were considered significant (∗p < 0.05, ∗∗p < 0.01, ∗∗∗p < 0.001, ∗∗∗∗p < 0.0001. “ns” means non-significance).

We sought to further improve the engraftment of HSPCs by exploiting the transient overexpression of CXCR4. We optimized a transient *in vitro* transcription (IVT) platform testing different 5′/3′ UTR sequences, poly-adenylation tail lengths, and capping (Figures S4A–S4D). Our starting construct, pVAX, contained an ARCA capping, followed by a Kozak sequence, a WPRE sequence, and a 60 bp polyA tail. The optimized pVAXi construct comprised an AG Cleancap, an Eif4 aptamer at the 5′ UTR, and a WPRE sequence at the 3′ UTR, followed by a 150-bp polyA tail. Modified uridine (pseudouridine) was incorporated during the mRNA synthesis, followed by high-performance liquid chromatography (HPLC) purification, necessary to alleviate an interferon response in the electroporated cells (Figures S4E–S4H). *CXCR4* mRNA electroporation led to an increase in cells expressing CXCR4 and its MFI in the bulk CD34^+^ population (Figures 4A and 4B) and in primitive HSPCs (CD34^+^CD38^−^CD90^+^; Figures 4C and 4D) for up to 3 days. Transmigration assays were used to determine the impact of CXCR4 overexpression on cell migration. HSPCs overexpressing CXCR4, including the more primitive subset, migrated more toward CXCL12, compared with control cells electroporated with *GFP* mRNA (Figures 4E and 4F; Figures S4I–S4L). We tested two isoforms of CXCR4, differing from 9 amino acids at the N terminus (Duquenne et al., 2014; Gupta and Pillarisetti, 1999). Isoform 1 led to a higher response to CXCL12 and was used for the subsequent experiments (Figures 4G–4I).

**Figure S5:**
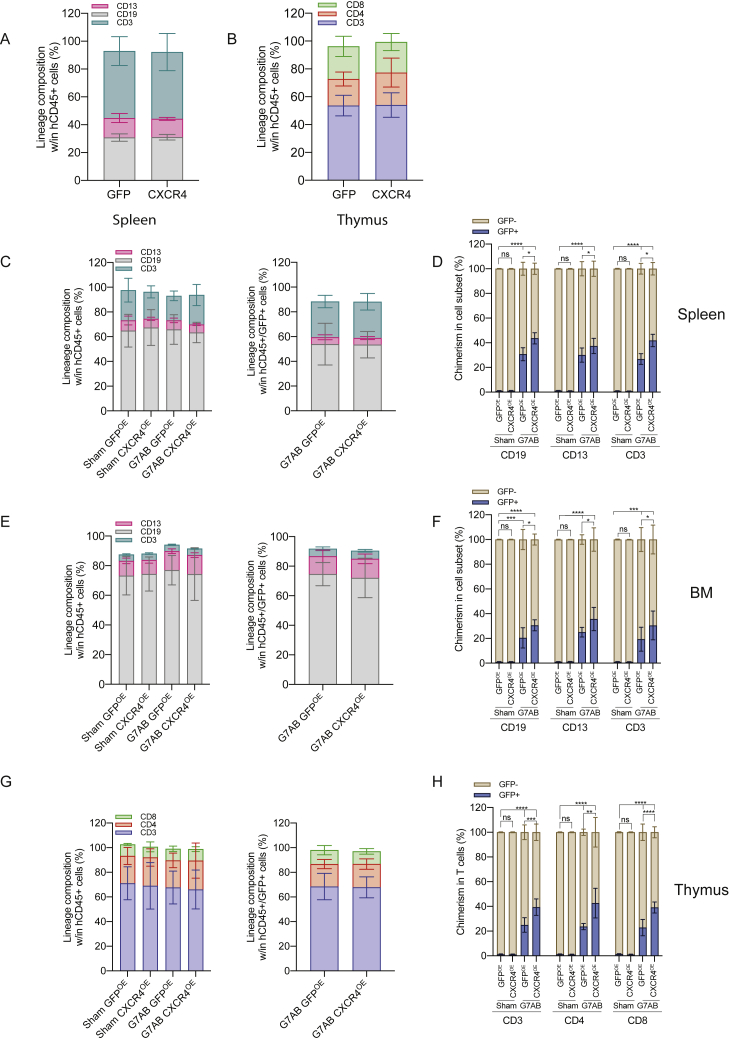
The transient overexpression of CXCR4 increases chimerism in the humanized context, following M-HSCT, related to Figure 4 (A and B) Myeloid and lymphoid cell composition of human CD45^+^ in the spleen (A) and thymus (B) of NSG mice, transplanted with CD34^+^ cells electroporated with *GFP* or *CXCR4* mRNA. The comparison of lineages between human CD45^+^ and GFP^+^ cells by a mixed-effects model (REML), followed by a post hoc analysis with Dunnett’s. (C, E, and G) Myeloid and lymphoid lineage composition of human CD45^+^ (left panel) and CD45^+^/GFP^+^ cells (right panel) in the spleen (C), BM (E), and thymus (G) after M-HSCT (as in Figure 3D) with CD34^+^ cells transiently overexpressing GFP or CXCR4 in NSGW41 mice. The comparison of lineages between human CD45^+^ and CD45^+^/GFP^+^ cells was performed by a mixed-effect model (REML), followed by a post hoc analysis with Dunnett’s. (D, F, and H) Chimerism of GFP^+^ cells was observed within CD19^+^, CD13^+^, and CD3^+^ cells in the spleen (D), BM (F), and within CD3^+^, CD4^+^, and CD8^+^ T cells in the thymus (H) after M-HSCT with CD34^+^ cells transiently overexpressing GFP or CXCR4 in NSGW41 mice. A mixed-effect model (REML), followed by a post hoc analysis with Tukey’s test (between groups) or by a post hoc analysis with Dunnett’s test (within groups). The results are mean ± SEM, with n ≧ 10. In all the analyses, p less than 0.05 were considered significant (∗p < 0.05, ∗∗p < 0.01, ∗∗∗p < 0.001, ∗∗∗∗p < 0.0001. “ns” means non-significance).

The impact of CXCR4 overexpression on engraftment was first evaluated in immunodeficient NOD-SCID-IL2Rg^−/−^ (NSG) mice. Cells transiently overexpressing CXCR4 yielded a larger human graft in the PB, reaching a median of 45% human chimerism compared with 35% of cells overexpressing control GFP (Figure 4J). The lineage distribution was similar in both groups in the PB, BM, spleen, and thymus (Figures 4K and 4L; Figures S5A and S5B). Interestingly, BM analysis showed an increased representation of the most primitive fraction of human HSPCs in mice transplanted with CXCR4 overexpressing cells (Figure 4M).

We next tested the potential advantage contributed by CXCR4 overexpression in niche re-colonization upon M-HSCT. NSGW41 mice stably engrafted with 3 × 10^5^ CD34^+^ cells were treated for mobilization, then infused with the outgrowth of 2 × 10^5^ cells, counted at day 1 post thawing, and transduced with GFP-LV and transiently overexpressing CXCR4 mRNA from the same donor as the original transplant. In parallel, the control mice were transplanted, after mobilization, with HSPCs transduced with GFP-LV and transiently overexpressing GFP mRNA (Figure 4N). The CXCR4 overexpressing cells efficiently outcompeted the mobilized HSPCs and established a stable chimerism in the human cell graft, whereas the control cells overexpressing GFP engrafted to a lower extent (Figures 4O–4Q). Furthermore, we confirmed that mobilization was indispensable to obtain chimerism with the newly infused cells, whether advantaged or not by CXCR4 overexpression, as observed by the lack of engrafted GFP^+^ cells in non-mobilized mice. By the end of the experiment, lineage composition was similar in mobilized and non-mobilized mice in the PB (Figure 4R), and the chimerism in each subset remained stable (Figure 4S). These results were confirmed in the spleen, BM, and thymus, where higher levels of GFP were detectable in mice transplanted with cells initially overexpressing CXCR4, without impacting the proportion of progenitors and differentiating cells within the different hematopoietic organs (Figures S5C–S5H). Secondary transplants of matched numbers of human CD34^+^ cells purified from the BM of primary transplanted mice revealed the presence of human GFP^+^ grafts in mice transplanted with cells harvested from mobilized groups only, proving the successful engraftment of LT-HSC following M-HSCT (Figure 4T). Moreover, the percentage of GFP^+^ cells in the secondary recipients was higher in the group transplanted with human cells from primary recipients of CD34^+^ cell transiently overexpressing CXCR4 versus cell transiently overexpressing control GFP mRNA, emphasizing the advantage of using an engraftment enhancer (Figure 4U).

### M-HSCT confers a clear advantage to gene-edited cells when paired with an engraftment enhancer

**Figure S6:**
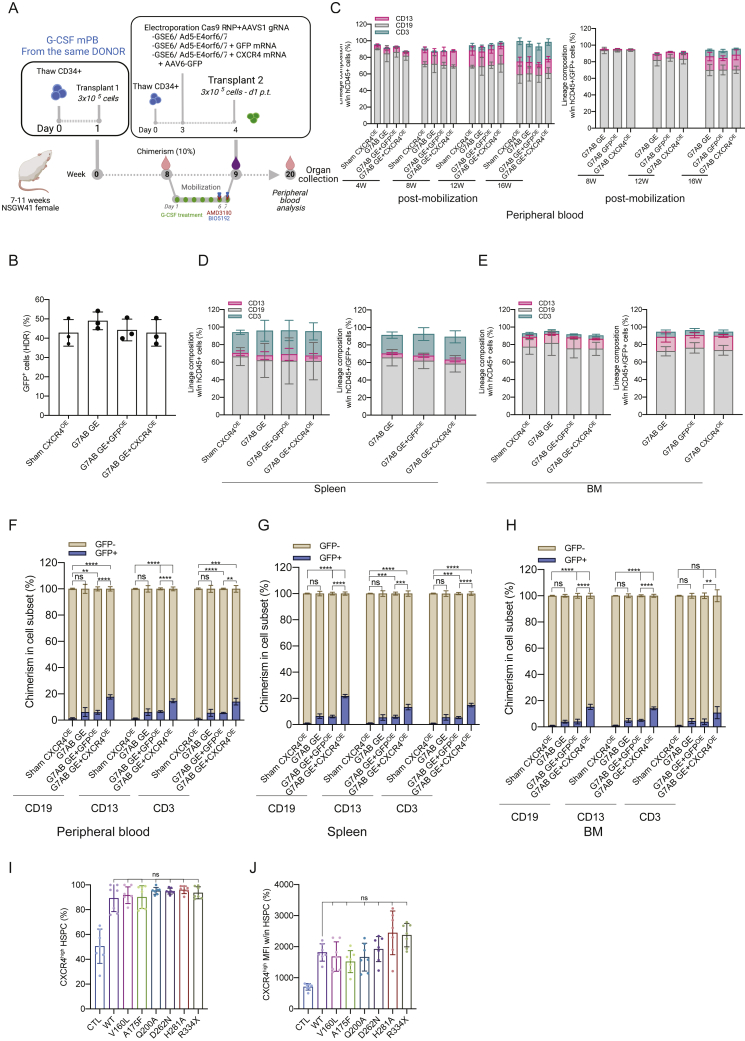
M-HSCT confers a significant advantage to gene-edited cells when paired with an engraftment enhancer, related to Figure 5 (A) Schematic illustration of competitive transplantation, after G7AB mobilization, between human resident cells (initially transplanted with 3 × 10^5^ CD34^+^ G-CSF mPB cells, counted on d1 p.t.) and newly transplanted CD34^+^ G-CSF mPB gene-edited cells (an outgrowth of 3 × 10^5^ CD34^+^ cells, counted on d1 p.t., and transplanted on d4 p.t.) in NSGW41 mice. (B) HDR efficiency in edited cells (GFP^+^) on the bulk CD34^+^ population, assessed *in vitro*, 15 days post electroporation. (C) The reconstitution of myeloid and lymphoid lineages over time of human CD45^+^ (left panel) and CD45^+^/GFP^+^ cells (right panel) in PB after M-HSCT with CD34^+^ cells gene edited as in main Figure 5A, in NSGW41 mice. The comparison of lineages between human CD45^+^ and CD45^+^/GFP^+^ cells was performed at the last time point by a mixed-effects model (REML), followed by a post hoc analysis with Dunnett’s test (within groups). (D and E) Myeloid and lymphoid lineage composition of human CD45^+^ (left panel) and CD45^+^/GFP^+^ cells (right panel) in the spleen (D) and BM (E) after M-HSCT with CD34^+^ cells gene edited as in main Figure 5A, in NSGW41 mice. The comparison of lineages between human CD45^+^ and CD45^+^/GFP^+^ cells was performed by a mixed-effect model (REML), followed by a post hoc analysis with Dunnett’s test (within groups). (F–H) Chimerism of GFP^+^ cells was observed within CD19^+^ B cells, CD13^+^ myeloid cells, and CD3^+^ T cells at the end of the experiment in the PB (F), spleen (G), and BM (H) after M-HSCT with CD34^+^ cells gene edited as in main Figure 5A, in NSGW41 mice. A mixed-effect model (REML), followed by a post hoc analysis with Tukey’s test (between groups) or by post hoc analysis with Dunnett’s test (within groups). (I) The percentage of CXCR4^high^ cells in HSPCs (CD34^+^CD133^+^CD90^+^), electroporated with *CXCR4* variants mRNA. A Kruskal-Wallis test, followed by a post hoc analysis with Dunn’s test. (J) CXCR4^high^ MFI in HSPCs (CD34^+^CD133^+^CD90^+^), electroporated with *CXCR4* variants mRNA. A Kruskal-Wallis test, followed by a post hoc analysis with Dunn’s test. The results are mean ± SEM, with n ≧ 8 for data collected *in vivo* on NSGW41 mice, with n ≧ 5 for the data collected *in vivo* on NSG mice and n ≧ 5 for data collected *in vitro* (3 different donors). In all the analyses, p less than 0.05 were considered significant (∗p < 0.05, ∗∗p < 0.01, ∗∗∗p < 0.001, ∗∗∗∗p < 0.0001. “ns” means non-significance).

**Figure 5:**
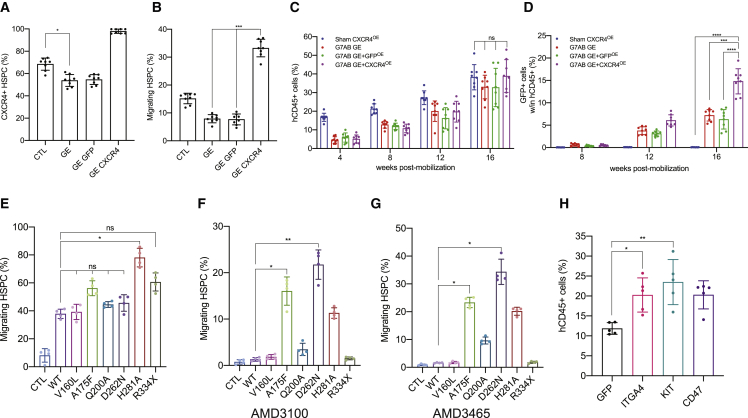
M-HSCT confers a significant advantage to gene-edited cells when paired with an engraftment enhancer (A and B) The percentage of CXCR4^+^ cells in HSPCs (CD34^+^CD133^+^CD90^+^) (A) and of migrating HSPCs (B), following gene editing (GE; Cas9 RNA, AAVS1 sgRNA, and AAV6-GFP) combined or not with *GFP* (GE GFP) or *CXCR4* (GE CXCR4) mRNA. (C and D) Long-term follow-up of human CD45^+^ (C) and CD45^+^/GFP^+^ (D) cells in the PB after M-HSCT (as in Figure 3D) with CD34^+^ cells gene edited as in (A), in NSGW41 mice. (E–G) The percentage of migrating HSPCs without treatment (E), in the presence of AMD3100 (F) or AMD3465 (G), electroporated with CXCR4 variants. (H) The percentage of human CD45^+^ in the PB of NSG mice at 16 weeks, following the transplantation of CD34^+^ cells electroporated with *GFP*, *ITGA4*, *KIT*, and *CD47* mRNA. The results are mean ± SEM, with n ≧ 8 biological replicates for data collected *in vivo* on NSGW41 mice, with n ≧ 5 biological replicates for data collected *in vivo* on NSG mice and n ≧ 4 for data collected *in vitro* (3 biological replicates and 1 technical replicate). In all the analyses, p less than 0.05 were considered significant (∗p < 0.05, ∗∗p < 0.01, ∗∗∗p < 0.001, ∗∗∗∗p < 0.0001. “ns” means non-significance).

*Ex vivo* gene correction in autologous HSCT may decrease engraftment efficiency, especially when achieved by gene editing (Schiroli et al., 2019). First, we monitored the expression of CXCR4 in cells edited for the site-specific integration of a GFP-expressing cassette into the adeno-associated virus integration site 1 (AAVS1) safe harbor locus using a recently optimized protocol. The CD34^+^ cells were electroporated with Cas9 ribonucleoprotein (RNP) assembled with sgRNA targeting AAVS1 and editing enhancers (GSE56/Ad5-E4orf6/7) and immediately transduced with a repair template carrying AAV6-GFP vector (Figure S6A). CXCR4 expression was decreased in gene-edited cells as compared with electroporated-only cells (Figure 5A). To counteract this adverse impact on CXCR4, we co-electroporated cells with *CXCR4* mRNA combined with all the gene-editing machinery. The control counterparts received *GFP* mRNA with the same gene-editing machinery. Although gene-edited cells with or without GFP overexpression had decreased migration potential, gene-edited HSPCs overexpressing CXCR4 showed higher migration potential in an *in vitro* migration assay, even when compared with electroporated-only control cells (Figure 5B). We next tested whether CXCR4 overexpression could provide an engraftment advantage to edited cells. Although the chimerism level of edited cells (stably expressing GFP) only reached a median of 5% for the standard treatment, the addition of *CXCR4* mRNA allowed 3-fold enhanced engraftment reaching a median of 15% (Figures 5C and 5D). Intriguingly, because homology directed repair (HDR) efficiency was around 40% as measured in the more primitive subset *in vitro* (Figure S6B), the total chimerism reached *in vivo* by cells edited together with *CXCR4* mRNA would correspond to the levels reached by LV-transduced cells and electroporated with *CXCR4* mRNA in the previous experiment (40% GFP for cells transduced by LV to 90%). This result supports our previously reported finding that editing with optimized enhancers contains the impact of the procedure on HSPC repopulation properties (Ferrari et al., 2020). Lineage proportions within PB, BM, and spleen were similar for edited and non-edited cells, with or without the initial overexpression of CXCR4 (Figures S6C–S6H).

Overall, these findings show the portability of our strategy across different genetic engineering strategies and the feasibility of establishing a human hematopoietic graft comprising a fraction of edited cells sufficient for providing therapeutic benefits in diseases such as HIGM-1, without resorting to any preconditioning.

Whereas the results of M-HSCT as described here are already promising, multiple strategies could be used to improve its efficacy further. We tested different variants of CXCR4 (Ieranò et al., 2009; McDermott et al., 2011; Rosenkilde et al., 2004, 2007), which all led to an increased surface overexpression of CXCR4 and were able to endow HSPCs with a further migration advantage (Figure 5E; Figures S6I and S6J). Interestingly, two of these variants were resistant to AMD3100 and even more to AMD3465, while keeping an efficient response to CXCL12, as shown by the migration of overexpressing cells in the presence of CXCR4 antagonists (Figures 5F and 5G). These variants could be used in the context of M-HSCT to further enhance the competitive advantage of infused cells over the mobilized cells and, conceivably, the extent of chimerism established.

Moreover, other molecules could be exploited besides CXCR4 for endowing the infused cells with a transient competitive advantage. As the first proof of principle, we showed that the overexpression of *KIT*, *ITGA4*, and *CD47* mRNA could all provide an *in vivo* engraftment advantage (Figure 5H), similar to or even higher than that shown for CXCR4, to infused human cells in the context of M-HSCT, supporting its versatility and broad potential when coupled to mRNA-based genetic engineering of the administered cells.

## Discussion

Here, we provide evidence that HSPC mobilization might be successfully exploited in HSCT, taking advantage of a window of opportunity opened at the peak of mobilization when donor cells might effectively outcompete those in the circulation for repopulation of the depleted BM niches. The competitive advantage of donor cells results from the rescue during *ex vivo* culture of a detrimental impact of mobilizers on HSPCs and might be further enhanced by the transient overexpression of engraftment effectors. We present a proof of principle for the therapeutic efficacy of M-HSCT in a mouse model of HIGM-1, although using healthy BM cells as a surrogate of autologous genetically corrected ones, and further developed it using human hematochimeric mouse models showing its applicability to human HSPCs and its versatility when coupled to different genetic engineering strategies, such as gene replacement and gene editing. Overall, our findings encourage the eventual disposal of conventional genotoxic conditioning when designing autologous HSPC-GT and should pave the way to its broader and safer use in a relevant number of inherited diseases.

Whereas it is well established that mobilized HSPCs can home and successfully engraft in conditioned recipients, the processes underlying reconstitution to full niche occupancy are less well understood (Bhattacharya et al., 2009). Whether and to what extent residual HSPCs in the BM and those still in the circulation (in our case) compete with the infused ones in these processes is unknown. Infused HSPCs can be trapped in different non-hematopoietic locations and become phagocytosed, decreasing, even more, the effective therapeutic dose administered and engraftment (Ratajczak and Suszynska, 2016; Szumilas et al., 2005). Our findings shed some light on the dynamics and source of such competition. The short window of time used for M-HSCT suggests that early niche occupancy by the cells in the circulation prevents repopulation from the residual ones in the BM. This hypothesis is also supported by recent reports that describe the possibility of editing HSPCs *in vivo*, which requires prior cell mobilization and thus suggest that egressed cells from the niche may contribute to long-term hematopoiesis (Li et al., 2021)

Our strategy is built-in the process of *ex vivo* HSPC engineering. The *ex vivo* culture allows the recovery of surface molecules crucial for homing/engraftment and whose expression has been lowered by the mobilization protocol (Lévesque et al., 2003a). On the other hand, when genetic engineering has a detrimental impact on the engraftment or repopulation potential of the treated HSPCs, as reported for gene editing (Ferrari et al., 2020), HSPC fitness can be enhanced by the transient overexpression of molecular targets involved in the homing process concomitantly to the genetic modification. Conceivably, this approach would also be compatible with a short process time, in which mobilized cells immediately undergo genetic engineering and exchange. Of note, in the clinical setting where HSPC harvest and M-HSCT were to be performed almost concurrently, mobilized cells in the recipient would have been depleted from the circulation at the time of infusion of the engineered ones, thus, further reducing competition for engraftment.

Our findings build upon the recent advances in mRNA engineering, which by using modified nucleotides and optimized design allow increasing the time and extent of expression in transfected cells without eliciting innate recognition and detrimental responses (Tusup et al., 2019). Moreover, the adoption of an “mRNA-only” platform safeguards against the risk of even sporadic integration of the effector transgene into the cellular genome, thus, allowing the safe capture of powerful gain-of-function effectors to our purpose. These include homing receptors such as CXCR4 (Kahn et al., 2004), adhesion molecules such as ITGA4 (Vermeulen et al., 1998), and inhibitors of professional phagocytosis such as CD47 (Jaiswal et al., 2009) but might conceivably be extended to other genes involved in pathways such as self-renewal (Liesveld et al., 2020; Seita and Weissman, 2010). Moreover, the mRNA-based delivery of engraftment enhancers can be easily incorporated into the *ex vivo* gene-editing process, without increasing its overall time or burden, except for the total amount of transfected mRNA, which must be comprised within the limits of cell tolerability.

Although the M-HSCT protocols described here comprise some reagents not yet approved for clinical use, several new mobilizers are emerging for clinical testing, such as GRO-Beta (CXCL2), which antagonizes CXCR2 (Fukuda et al., 2007; King et al., 2001). As these new reagents become clinically applicable, they may allow even more HSPC mobilization and, thus, more effective exchange, possibly allowing the removal of G-CSF treatment. In this case, BM niches may be better preserved, allowing faster hematopoietic reconstitution by the gene-corrected cells. On the other hand, removing G-CSF may fail to induce the surface modifications that decrease the competitiveness of mobilized cells, in which case the use of engraftment enhancers might be necessary. It is likely that our strategy might be relatively blind to the nature and mechanisms of action of the mobilizers used, provided that efficient HSPC depletion is achieved, and the newly infused cells are suitably engineered to outcompete the residual ones. Although we have not specifically investigated here the toxicity profile of our strategy, we expect it to be in line with that associated with the clinical use of mobilizers, which have shown an excellent safety record with only minimal adverse effects reported (Mueller et al., 2013; Pulsipher et al., 2009). Moreover, given the low toxicity associated with the mobilization protocol, the serial administration of corrected cells from a stored cell product for multiple administrations following mobilization cycles can be envisioned to further increase engraftment.

Of note, the use of G-CSF and AMD3100 as preparative regimens for patients with severe combined immunodeficiency undergoing HSCT has been tested and reported as inefficient (Dvorak et al., 2014). However, the percentage of mobilized CD34^+^ cells in the PB was suboptimal highlighting a limited BM vacancy, which might explain the low/absent chimerism (Table 2). Moreover, donor CD34^+^ cells were mobilized but not cultured *ex vivo*, thus potentially bearing a lower homing and engraftment potential as shown here and supporting the requirement for the *ex vivo* engineering step presented in our work.

**Table 2:** Estimated chimerism following M-HSCT in pediatric patients

Estimated total CD34^+^ cells in the BM	counts of WBCs in the human BM (mL)	3.05E+7	Canarutto et al. (2021)
	percentage of CD34^+^ within WBCs in BM	3.8	Canarutto et al. (2021)
	estimated total CD34^+^ in BM (mL)	1.16E+6	Canarutto et al. (2021)
	total BM (mL, 15-kg child)	1,600	Orkin et al. (2015)
	total CD34^+^ in BM (15-kg child)	1.85E+9	Orkin et al. (2015)
Estimated mobilized CD34^+^ cells	collected CD34^+^ after G-CSF+Plerixafor (cells/kg)	3.70E+7	Canarutto et al. (2021)
	total collected CD34 (15-kg child)	5.55E+8	Canarutto et al. (2021)
	BM vacancy (%)	29.93	N/A
Estimated chimerism following infusion of corrected cells	CD34^+^ required for autologous HSCT-GT (kg) (upper range)	3.50E+7	Canarutto et al. (2021)
	total CD34^+^ required (15-kg child)	5.25E+8	Canarutto et al. (2021)
	percentage of estimated chimerism	14.5	N/A
Modeling to clinical trial NCT01182675 (Dvorak et al., 2014)	unique patient number	#1,056	#1,535
	counts of WBCs in human PB (/L)	5.00E+9	1.00E+10
	percentage of CD34^+^ within WBCs in PB	1	1.5
	estimated CD34^+^ in PB (L)	5.00E+7	1.00E+8
	age	6 years	2 months
	estimated weight for age (kg)	15	5
	estimated blood volume based on 70 mL/kg (L)	1.05	0.35
	estimated total CD34^+^ in PB	5.25E+7	3.50E+7
	BM vacancy (%)	2.83	1.89
	infused CD34^+^	2.40E+8	2.00E+8
	percentage of obtained chimerism	0	0
	percentage of estimated chimerism	2.3	1.6

We estimated the total CD34^+^ cells available in the BM and the median number of mobilized CD34^+^ cells following mobilization with G-CSF and AMD3100, according to reported findings (Canarutto et al., 2021; Orkin et al., 2015) and then determined the expected extent of BM vacancy. Based on the assumptions that: (1) mobilized HSPCs found in the circulation account for all BM-egressed cells and (2) mobilized and infused HSPCs compete equally, we estimate that upon infusion of 35 × 10^6^ CD34^+^/kg, a dose close to the upper range of those used in the clinic, one could reach a chimerism of 15%. We then performed the same calculation using data from a single (to the best of our knowledge) clinical trial testing transplantation post mobilization in a pediatric patient (Dvorak et al., 2014) and estimated a myeloid chimerism of 2%, close to the assay sensitivity. However, the reported chimerism was 0%, which might be due to the paired effect of (1) low mobilization efficiency (a 5- to 10-fold difference from optimal protocols reported in Canarutto et al., 2021 and personal communication), (2) the non-optimal window of infusion, missing the peak of mobilization, and (3) the possible low homing and engraftment potential of donor cells, which were harvested upon mobilization but not cultured *ex vivo*.

Interestingly, increased donor engraftment and event-free survival following the addition of G-CSF/AMD3100 to the conditioning regimen of Wiskott-Aldrich syndrome patients undergoing allogenic HCT have been reported (Balashov et al., 2018). Similarly, mixed chimerism after G-CSF/AMD3100 administration in addition to a nonmyeloablative conditioning regimen in patients with acute myelogenous leukemia has been reported (Konopleva et al., 2015), further supporting the contention that mobilization may enhance the engraftment of infused cells.

Looking forward, our strategy could be first tested and developed in the context of HSCT-GT, given that autologous cells do not need to overcome immune barriers in the recipient and that a mixed chimerism ranging around 30% might be sufficient for therapeutic benefit in many diseases that are candidates for HSPC-GT (Zimmerman and Shenoy, 2020). These include most primary immunodeficiencies but might also extend to hemoglobinopathies and some lysosomal storage disorders. Indeed, we provide here the proof of principle of the phenotypic rescue of HIGM-1 by M-HSCT in the mouse model, albeit using WT cells as a surrogate of edited cells. Allogeneic HSCT is the only curative treatment currently available for this disease. However, matched donors are not always available, and the procedure is associated with a high risk of graft rejection, GvHD, infections, liver failure, and death (Ferrua et al., 2019). Indeed, despite its curative potential, HSCT has little impact on ameliorating survival of HIGM-1 patients. Thus, therapeutic alternatives to treat patients safely and more effectively for whom HSCT is too risky are strongly needed. M-HSCT with autologous cells corrected by gene editing might represent a promising option, where the use of engraftment enhancers might also compensate for the limited efficiency of HDR in primitive HSPCs (Vavassori et al., 2021).

If successful, the combination of mobilization and increased engraftment efficiency investigated in our studies might provide an innovative way to entirely bypass the requirement for chemo/radiotherapy in HSPC-GT, conferring long-term therapeutic benefits with considerably less risk and long-term toxicity to patients. Novel condition regimes based on selective immunodepletion might also fit our strategy, as matching engraftment enhancers could be used, such as a mutant KIT not recognized by the anti-KIT antibodies/immunotoxin used for depletion, according to the model demonstrated here for the AMD3100-resistant CXCR4 variant. The combination of our engraftment enhancement strategy with an immunotoxin-based strategy or low-dose chemotherapy may eventually further broaden its efficiency and applicability beyond autologous HSCT.

### Limitations of the study

Our study of human HSCT has been necessarily performed in hematochimeric mouse models, which represent the most adopted model for testing new experimental manipulations of human HSPC. This choice imposes some limitations on the translation of the results in a future clinical setting. The murine BM niche may not fully represent the human architecture and local factors may have lower specific activity toward human cells and/or different biodistribution. Indeed, NSGW41 hosts were required to demonstrate an efficient exchange of infused human HSPC as they provide for less competition from mouse resident cells and compensate for the lower specific activity of murine factors on human HSPC. However, when using engraftment enhancers our strategy also worked in standard NSG hosts. We do expect that our findings might still be predictive of a fully human setting because of the following: (1) the mobilization yields resemble those obtained in the clinic (50–100 CD34^+^/μL mobilized) and (2) the parallel findings were obtained with mouse and human HSPC. Non-human primate studies might, however, be required for further validation and dose adjustment of our strategy before contemplating its clinical testing.

## STAR★Methods

### Key resources table

**Table:** 

REAGENT	SOURCE	IDENTIFIER
**Antibodies**		

Anti-human CD3	BD Biosciences	Cat# 555335; RRID:AB_398591
Anti-human CD3	BioLegend	Cat# 980004; RRID:AB_2632620
Anti-human CD3	BioLegend	Cat #300316; RRID:AB_314052
Anti-human CD4	BD Phosflow	Cat# 558116; RRID:AB_397037
Anti-human CD13	BD Biosciences	Cat# 557454; RRID:AB_398624
Anti-human CD13	Invitrogen	Cat# MHCD1304; RRID:AB_1730807
Anti-human CD19	BD Biosciences	Cat# 345789; RRID:AB_2868815
Anti-human CD19	BioLegend	Cat# 302216; RRID:AB_314246
Anti-human CD19	Miltenyi Biotec	Cat# 130-113-172; RRID:AB_2725999
Anti-human CD33	BD Biosciences	Cat# 333946; RRID:AB_399961
Anti-human CD33	Miltenyi Biotec	Cat# 130-099-485; RRID:AB_2660351
Anti-human CD34	BD phosflow	Cat# 348811; RRID:AB_2868855
Anti-human CD34	Miltenyi Biotec	Cat# 130-095-393; RRID:AB_10827793
Anti-human CD34	Miltenyi Biotec	Cat# 130-090-954; RRID:AB_244349
Anti-human CD38	BioLegend	Cat# 356614; RRID:AB_2562183
Anti-human CD38	Miltenyi Biotec	Cat# 130-113-428; RRID:AB_2733230
Anti-human CD45	BD Biosciences	Cat# 641417; RRID:AB_2800453
Anti-human CD45	BioLegend	Cat# 304029; RRID:AB_2174123
Anti-human CD45	BioLegend	Cat# 304016; RRID:AB_314404
Anti-human CD45	BioLegend	Cat# 304008; RRID:AB_314396
Anti-human CD45	Miltenyi Biotec	Cat# 130-091-230; RRID:AB_244233
Anti-human CD45RA	BioLegend	Cat# 304112; RRID:AB_314416
Anti-human CD45RA	BioLegend	Cat#304128; RRID:AB_10708880
Anti-human CD47	BD Biosciences	Cat# 563760; RRID:AB_2744414
Anti-human CD47	Miltenyi Biotec	Cat# 130-101-407; RRID:AB_2658401
Anti-human CD49d	BioLegend	Cat# 304304; RRID:AB_314430
Anti-human CD90	BD Biosciences	Cat# 555596; RRID:AB_2868855
Anti-human CD90	BD Biosciences	Cat# 559869; RRID:AB_398677
Anti-human CD117	BioLegend	Cat# 313212; RRID:AB_893222;
Anti-human CD184	Miltenyi Biotec	Cat# 130-109-844; RRID:AB_2655771
Anti-human CD184	Miltenyi Biotec	Cat# 130-117-519; RRID:AB_2734059
Anti-human CD184	Miltenyi Biotec	Cat# 130-109-845; RRID:AB_2655775
Anti-human CD184	ThermoFisher Scientific	Cat#TA591094
Anti-human CD184	ThermoFisher Scientific	Cat#TA591092
Anti-human CD133/2	Miltenyi Biotec	Cat#170-070-702; RRID:AB_10831361
Human Fc Block	Miltenyi Biotec	Cat# 130-059-901; RRID:AB_2892112
Anti-mouse CD3	BD Biosciences	Cat# 555275; RRID:AB_395699
Anti-mouse CD3	BD Biosciences	Cat# 555275; RRID:AB_395699
Anti-mouse CD3	BD Biosciences	Cat# 557596; RRID:AB_396759
Anti-mouse CD4	BD Biosciences	Cat# 558107; RRID:AB_397030
Anti-mouse CD4	BD Biosciences	Cat# 553051; RRID:AB_398528
Anti-mouse CD8	eBioscience	Cat# 47-0081-82; RRID:AB_1272185
Anti-mouse CD11b	BD Biosciences	Cat# 553312; RRID:AB_398535
Anti-mouse CD19	BioLegend	Cat# 115520; RRID:AB_313655
Anti-mouse CD19	BioLegend	Cat# 115526; RRID:AB_493341
Anti-mouse CD24	BD Biosciences	Cat# 562360; RRID:AB_11151895
Anti-mouse CD25	BD Biosciences	Cat# 551071; RRID:AB_394031
Anti-mouse CD44	BD Biosciences	Cat# 563970; RRID:AB_2738517
Anti-mouse CD45.1	BD Biosciences	Cat# 553776; RRID:AB_395044
Anti-mouse CD45.1	BD Pharmingen	Cat# 553775; RRID:AB_395043
Anti-mouse CD45.2	BD Pharmingen	Cat# 552950; RRID:AB_394528
Anti-mouse CD45.2	BioLegend	Cat# 109820; RRID:AB_492872
Anti-mouse CD45R/B220	BioLegend	Cat# 103224; RRID:AB_313007
Anti-mouse CD48	BioLegend	Cat# 103418; RRID:AB_756140
Anti-mouse CD48	BioLegend	Cat# 103406; RRID:AB_313021
Anti-mouse CD62L	BD Biosciences	Cat# 553152; RRID:AB_398533
Anti-mouse CD69	Fisher Scientific	Cat# 12-0691-81, RRID:AB_465731
Anti-mouse CD117	BD Pharmingen	Cat# 553356; RRID:AB_398536
Anti-mouse CD117	eBioscience	Cat# 47-1171-82; RRID:AB_1272177
Anti-mouse CD150	BioLegend	Cat# 115910; RRID:AB_493460
Anti-mouse CD184	ThermoFisher Scientific	Cat# 14-9991-82
Anti-mouse GL7	eBioscience	Cat# 50-5902-82; RRID:AB_2574252
Anti-mouse Lineage Cocktail	BioLegend	Cat# 133303; RRID:AB_1595553
Live/dead	Life Technologies	Cat# L-34959
Anti-mouse Ly6A/E (Sca1)	BD Biosciences	Cat# 558162; RRID:AB_647253
Anti-mouse Ly6A/E (Sca1)	BioLegend	Cat# 108120; RRID:AB_493273
Mouse Fc Block	BD Biosciences	Cat# 553142; RRID:AB_394657
Anti-mouse PNA	Vector Laboratories	Cat# FL-1071; RRID:AB_2315097
Anti-mouse TCRbeta	BioLegend	Cat# 109211; RRID:AB_313434

**Chemicals, peptides, and recombinant proteins**		

5-Methoxyuridine-5'-Triphosphate	TriLink Biotechnologies	Cat# N-1093-10
5-Methylcytidine-5'-Triphosphate	TriLink Biotechnologies	Cat# N1014-10
ACK Lysing Buffer	ThermoFisher	Cat# A1049201
Alt-R Cas9 Electroporation Enhancer	Integrated DNA Technologies	Cat# 1075916
Alt-R CRISPR-Cas9 crRNA	Integrated DNA Technologies	Ferrari et al. 2021a
sNLS-SpCas9-sNLS Nuclease	Aldevron	Cat# 9212-5MG
AMD3100	Genzyme (Sanofi)	Mozobil
AMD3465	Sigma-Aldrich	Cat# SML1433-25MG
BIO5192	R&D System	Cat# 5051/10
CXCL12	PeproTech	Cat# 300-28A
CleanCap AG	Tebu-Bio	Cat#N-7113-5
G-CSF (Lenograstim)	Italfarmaco	Myelostim
hFlt3-L	PeproTech	Cat# 300-19
hSCF,	PeproTech	Cat# 300-07
hTPO	Tebu-bio	Cat# 300-18-0100
Imject Alum Adjuvant	ThermoFisher Scientifi	Cat# 77161
mFLT3-L	PeproTech	Cat# 250-31L
mSCF	PeproTech	Cat# 315-02
mTPO	PeproTech	Cat# 315-14
PGE2	Cayman Chemical Company	Cat# 39746-25-3
Pseudouridine-5'-Triphosphate	TriLink Biotechnologies	Cat# N-1019-10
SR1	BioVision	Cat# 1967-1
TNP-KLH (Keyhole Limpet Hemocyanin)	LGC Biosearch Technologies	Cat# T-5060
UM171	StemCell Technologies	Cat# 72914

**Critical commercial assays**		

5X MEGAscript T7 kit	Life Technologies	Catt# AMB1334-5
Apoptosis Detection kit	BD Pharmingen	Cat# 559763
Lineage Cell Depletion Kit	Miltenyi Biotec	Cat# 130-090-858
Mouse Cell Depletion Kit	Miltenyi Biotec	Cat# 130-104-694
Mouse pro MMP9 ELISA Kit	Thermo Scientific	Cat# EMMP9
Mouse SDF-1 alpha / CXCL12 alpha ELISA Kit, for serum, plasma, and cell culture	Sigma-Aldrich	Cat# RAB0125-1KT
P3 Primary Cell 4D-Nucleofector X Kit	Lonza	Cat# V4XP-3032
TaqMan Gene Expression Assay	ThermoFisher Scientifi	Cat# 4331182

**Experimental models: Cell lines**		

Human: G-CSF mPB CD34+ HSPCs	AllCells	N/A
Human: G-CSF/Mozobil mPB CD34+ HSPCs	AllCells	N/A
Human: HEK293T	ATCC	Cat# CRL-3216

**Experimental models: Organisms/strains**		

C57Bl/6 Ly45.1	Charles River Laboratory.	Cat# 494
C57Bl/6 Ly45.2	Charles River Laboratory.	Cat# 027
Cd40lg-/- (B6.129S2-Cd40lgtm1Imx/J)	Jackson Laboratory	Cat# 002770; RRID:IMSR_JAX:002770
NSG	Charles River Laboratory	Cat# 614
NSGW41	Jackson Laboratory	Cat# 026497; RRID:IMSR_JAX:026497

**Other**		

Micro-osmotic pump model 1003D	Alzet	Cat# 0000289
Micro-osmotic pump model 1007D	Alzet	Cat# 0000290
Transwell Permeable Support	Costar	Cat# 3421

### Resource availability

#### Lead contact

Please direct requests for resources and reagents to lead contact: Luigi Naldini (naldini.luigi@hsr.it).

#### Materials availability

Plasmids generated in this study are available from the lead contact upon request.

#### Data and code availability

• All data reported in this paper will be shared by the lead contact upon request.
• This paper does not report original code.
• Any additional information required to reanalyze the data reported in this work paper is available from the lead contact upon request.

### Experimental model and subject details

#### Mice

C57Bl/6 Ly45.1, C57Bl/6 Ly45.2 mice were purchased from Charles River Laboratory. *Cd40lg*^*-/-*^ (B6.129S2-Cd40lgtm1Imx/J), humanized NSG or NSGW41 mice were purchased from The Jackson Laboratory and maintained in specific-pathogen-free (SPF) conditions. The procedures involving animals were designed and performed with the approval of the Animal Care and Use Committee of the San Raffaele Hospital (IACUC #818 for *Cd40lg*^*-/-*^, #876 for NSGW41 and NSG) and communicated to the Italian Ministry of Health and local authorities according to Italian law.

Eight- to twelve- weeks old female mice were used for the experiments.

#### Cell line and primary cells

HEK293T cells were cultured in Iscove’s modified Dulbecco’s medium (Corning) supplemented with 10% heat-inactivated fetal bovine serum (Euroclone), 100 IU.ml^−1^ penicillin, 100 μg.ml^−1^, streptomycin and 2% glutamine.

G-CSF mPB CD34^+^ HSPCs and G-CSF/Mozobil mPB CD34^+^ HSPCs were purchased from Mobilized Leukopak (AllCells) according to TIGET-HPCT protocol approved by the San Raffaele Institute Bioethical Committee and purified with the CliniMACS CD34 Reagent System (Miltenyi Biotec) according to the manufacturer’s instructions. HSPCs were seeded at the concentration of 1×10^6^ cells per ml in serum-free StemSpan medium (StemCell Technologies) supplemented with 100 IU.ml^−1^ penicillin, 100μg.ml^−1^ streptomycin, 2% glutamine, 300ng.ml^−1^ hSCF, 300ng.ml^−1^ hFlt3-L, 100ng.ml^−1^ hTPO, 1μM SR1, 35nM UM171 and 10μM PGE2 (except when a subsequent transduction is planned). All cells were cultured in a 5% CO2 humidified atmosphere at 37 °C. *In vivo*, the human HSPC population was defined as CD34^+^CD38^-^CD90^+^, and *in vitro* as CD34^+^CD133^+^CD90^+^.

### Method details

#### Murine HSPC transplantation studies

Donor mice between 6 and 10 weeks of age were euthanized by CO_2_, and BM cells were retrieved from femurs, tibias, and humeri. HSPCs were purified by Lin- selection using the mouse Lineage Cell Depletion Kit (Miltenyi Biotec) according to the manufacturer’s instructions. Cells were then cultured (for 2 hours or overnight) in serum-free StemSpan medium (StemCell Technologies) containing penicillin, streptomycin, glutamine, and a combination of mouse cytokines (20 ng/ml IL-3, 100 ng/ml SCF, 100 ng/ml Flt-3L, 50 ng/ml TPO all from PeproTech), at a concentration of 2-5x10^6^ cells.ml^−1^. Purified Lin^-^ cells were transplanted at a total dose of 2x10^6^ cells/mouse into 8 to 12-week-old mobilized mice, three hours after the last injection of AMD3100 and/or Bio5192. Serial collections of blood from the retro-orbital vein were performed to monitor the hematological parameters and donor cell engraftment. At the end of the experiment, BM, thymus and spleen, and lymph nodes were harvested and analyzed.

#### ELISA

For ELISA performed on BM matrix, femurs and tibias were repeatedly flushed with 1mL of cold PBS. Cells were pelleted by centrifugation at 300 *g* for 10 minutes at 4°C, supernatant was then collected and stored at –80°C. ELISA has been performed following manufacturer instruction after a 1:100 dilution with 1x Assay Diluent for MMP9 (Thermo Scientific) or on undiluted BM matrix for CXCL12 (Sigma-Aldrich).

#### *In vivo* immunization and IgG quantification

Mice were immunized by intraperitoneal injection (i.p.) with 100 μg of TNP-KLH (Lgc Biosearch Technologies) in Imject Alum Adjuvant (1:2) (ThermoFisher Scientific), as described before (Vavassori et al., 2021). Serum was collected at day 0, 7, and 14 after immunization. Mice were boosted as described above on day 21, and serum was collected on day 7 after re-challenge. For IgG quantification, the concentration of antigen-specific IgGs in mouse sera was determined by an enzyme-linked immunosorbent assay (ELISA). Plates were coated with 100 μL/well of 5 μg/ml TNP-KLH in carbonate buffer. Following incubation, plates were washed three times in PBS containing 0.05% Tween20 (Sigma-Aldrich) (Wash Buffer). The plates were then blocked for 1 h using 100 μL/well of PBS containing 1% Bovine Serum Albumine (BSA), followed by a washing step, as described above. Serum samples were serially diluted in wash buffer and 100 μL/well of each diluted sample was added into the plate and incubated for 2 h at room temperature. For determination of the plate background optical density (OD) values, some wells were incubated with wash buffer alone. Following incubation, plates were washed and 100 μL/well of HRP-conjugated goat anti-mouse (Southern Biotech 1:10,000) was added and incubated for 1 h at room temperature. After washing, the plates were incubated for 5 min with 3,3’,5,5’0-tetramethyl benzidine (TMB, Sigma-Aldrich) substrate at room temperature. The reaction was stopped by the addition of 50 μL of 1 M H_2_SO_4_. The OD values at 450 nm were determined for each well using a Multiskan GO microplate reader (Thermo Fisher Scientific) and normalized to IgG1 standard curves. Results were expressed as mean of duplicate determinations.

#### LV vector production and titration

VSV.G-pseudotyped third-generation self-inactivating SINLV were produced by calcium phosphate transient transfection into 293T cells. 293T cells were transfected with a solution containing a mix of the LV genome transfer plasmid, bearing the expression cassette for GFP, the packaging plasmids pMDLg/pRRE, pMD2.VSV.G, pKRev(pILVV01) and pAdVantage (Promega). Medium was changed 14-16 hours after transfection and supernatant was collected 30 hours after medium change. LV-containing supernatants were passed through a 0.22 μm filter (Millipore) and transferred into sterile polyallomer tubes (Beckman) and centrifuged at 20,000 g for 120 min at 20° C (Beckman Optima XL-100KUltracentrifuge). LV pellet was dissolved in the appropriate volume of phosphate buffered saline (PBS) to allow 500X concentration. Concentrated vector stock was aliquoted and stored at -80°C. LV titer was determined by flow cytometry 4-5 days after LV transduction analysis or quantitative PCR, 10-14 days after LV transduction, as described (Milani et al., 2017).

#### mRNA IVT

*GFP, ITGA4, CXCR4, CD47* and *KIT* DNA coding RNA were synthetized (GeneArt, Thermo Fisher) using Homo Sapiens codon-optimized algorithm. A complementary *CXCR4* sequence was produced with reduced uridine content (dU). Coding sequences were subcloned in ‘pVax’ plasmids under the control of the following 5’ aptamer sequence: CapAG – eIF4G aptamer (GACTCACTATTTGTTTTCGCGCCCAGTTGCAAAAAGTGTCG) - Kozak sequence (CCACC) – start codon (ATG). Downstream the codon-optimized sequence follows a woodchuck hepatitis virus posttranscriptional regulatory element and a 120-bp polyA sequence.

For mRNA IVT, pVAX plasmids were linearized with SpeI (New England Biolabs) restriction enzyme and purified by phenol-chloroform extraction. mRNA was *in vitro* transcribed using the commercial 5X MEGAscript T7 kit (Thermo Fisher) and capped with 5mM of CleanCapAG (Trilink). Different modified nucleotides were used: 5-Methoxyuridine-5'-Triphosphate (moU), Pseudouridine-5'-Triphosphate (pU), 5-Methylcytidine-5'-Triphosphate (mC; Trilink) at a concentration of 7.5mM. mRNA was purified using RNeasy Plus Mini Kit (Qiagen) followed by high-performance liquid chromatography purification (ADS BIOTEC WAVE System) and Amicon Ultra- 15 (30 K) tube (Millipore) concentration. mRNA productions were aliquoted and stored at −80 °C. All RNA samples were analyzed by denaturing agarose gel electrophoresis to assess the quality and integrity.

#### Transduction

10^6^ CD34^+^ cells/ml were stimulated with 10μM PGE2 20 hours post-thawing. After 2 hours of pre-stimulation, cells were infected for 14 hours with LV-GFP at multiplicity of infection (MOI) 100. When necessary, cells were electroporated 14 hours post-transduction.

#### Electroporation

Cells were electroporated with 5μg of encoding mRNA, two days post-thawing. Transfections were performed using the 4D-NucleofectorTM System (Lonza) and following manufacturer’s instructions primary cells (P3 Primary Cell 4D-Nucleofector X Kit, program EO-100; Lonza). From 6h to day 10 after electroporation, target protein expression within HSPC subpopulations was evaluated by flow cytometry.

#### Gene editing of human HSPCs and analyses

For AAV6-based gene editing, 1×10^6^ CD34^+^ cells (mobilized with G-CSF) after 3 days of culture in the medium described above were washed with ten volumes of DPBS and electroporated using P3 Primary Cell 4D-Nucleofector X Kit and program EO-100 (Lonza). Cells were electroporated with RNPs at a final concentration of 2.5 μM together with 0.1 nmol of Alt-R Cas9 Electroporation Enhancer (Integrated DNA Technologies), according to the manufacturer’s instructions. AAV6 transduction was performed at a dose of 1×10^4^ vg per cell 15 min after electroporation. Additional mRNAs were added in the gene editing mixture as follows: (i) 3.5μg *GSE56/Ad5-E4orf6/7* (Fusion protein with P2A self-cleaving peptide) mRNA, (ii) 3.5μg *GSE56/Ad5-E4orf6/7* mRNA and 3.5μg *GFP* mRNA or *CXCR4* mRNA. Three and fifteen days after the editing procedure, cells were harvested to measure the percentage of cells expressing the GFP marker by flow cytometry and to extract gDNA for molecular analyses, as described (Ferrari et al., 2021c).

#### *In vitro* migration assay

Migration was performed using transwells permeable supports (Costar, 5μm polycarbonate membrane). Briefly, 2x10^5^ mPB CD34^+^ previously electroporated with target mRNA, were seeded in the upper chamber, in StemSpan medium with cytokines. The lower chamber was filled with 600 μl of StemSpan medium with cytokines, supplemented with recombinant CXCL12 (PeproTech, 125ng/ml). After 3 hours, migrating cells were recovered from the lower chamber and quantitatively evaluated on the BD Accuri™ C6 flow cytometer. For the migration inhibition experiment, Mozobil/AMD3100 and AMD3465 were added at 200 μM.

#### Flow cytometry

Immunophenotypic analyses were performed on fluorescence activated cell sorting FACS Canto II (BD Pharmingen) according to manufacturers’ instructions, equipped with DIVA Software and analyzed with the FSC express software (v. 6, 7, De Novo Software). 5×10^4^ - 2×10^5^ cells (from culture or mouse samples) were harvested, washed with PBS or MACS buffer (PBS pH 7.2, 0.5% BSA, 2mM EDTA), treated with fragment crystallizable (Fc) Receptor-Block (Miltenyi Biotec), when antibody stained, and then re-suspended in the buffer used for washing. Staining was performed in MACS buffer, incubating cells for 15 minutes at 4° C in dark with a mix of antibodies listed below, in a final volume of 100 μL. Sphero Rainbow Calibration Particles (Spherotech) beads were used to calibrate the instrument detectors, for consistent MFI measurement, for analysis performed at different times. Single stained and Fluorescence Minus One (FMO)-stained cells were used as controls. To stain the human CXCR4, an antibody targeting CXCR4 N-terminus epitope was used, except in the Figure S3D, where the human CXCR4 was stained with an antibody targeting CXCR4 ECL2 epitope.

LIVE/DEAD Fixable Dead Cell Stain Kit (Thermo Fisher) or 7-aminoactinomycin (Sigma-Aldrich) was included in the sample preparation for flow cytometry according to the manufacturer’s instructions to exclude dead cells from the analysis. Apoptosis analysis was performed on CD34^+^ cells one day after electroporation using Annexin V (Biolegend) and Apoptosis Detection kit with 7-Aminoactinomycin D (7AAD, BD Pharmingen) according to the manufacturers’ instructions. Percentages of live (7AAD^−^, AnnexinV^−^), early apoptotic (7AAD^−^, AnnexinV^+^) and late apoptotic (7AAD^+^, AnnexinV^+^) cells are reported.

For intracellular staining, surface antigens were stained prior to fixation and permeabilization steps, performed using the BD Cytofix/Cytoperm fixation/permeabilization Kit, according to the manufacturer’s instructions.

Blood samples were also analysed with the hemocytometer Sysmex KX-21N (Block scientific, Sysmex corporation) to quantify absolute numbers.

#### CD34^+^ HSPC xenotransplantation experiments in NSG and NSGW41 mice

For transplantation into sublethally irradiated (150–180 cGy) NSG mice, 3x10^5^ cord blood CD34^+^ cells, diluted in 200 μL of PBS, were injected intravenously 24 hours after electroporation (performed 48hours post-thawing).

For transplantation into NOD-B6-SCID Il2rγ-/- Kit(W41/W41) mice, 3x10^5^ G-CSF mPB CD34^+^ cells, diluted in 200 μL of PBS, were injected intravenously 48-72hours post-thawing. Once the human chimerism reached 10%, humanized NSGW41 mice were mobilized and transplanted with 1-3x10^5^ G-CSF mPB CD34^+^ cells, transduced (for stable overexpression of a fluorescent marker) the first day post-thawing and/or electroporated on the third day post-thawing, and transplanted on the fourth day. Mice were randomly distributed to each experimental group.

**Table:** 

# G-CSF mPB CD34	1^st^ transplant (Counted at day1)	2^nd^ transplant (Counted at day1)
Figure 3 Gene replacement	1x10^5^	1x10^5^ (Transplanted at day 3)
Figure 4 Gene replacement + Electroporation	1x10^5^	2x10^5^ (Transplanted at day 3)
Figure 5 Gene correction	1x10^5^	3x10^5^ (Transplanted at day 4)

Human CD45^+^ cell engraftment, cell lineages and/or GFP^+^ cells were monitored by serial collection of blood from the retro-orbital vein and, at the end of the experiment (>12 weeks after transplantation), BM, thymus and spleen were harvested and analysed by flow cytometry for end-point analyses.

BM was flushed with PBS 2% BSA and 50 μL were stained for surface markers. The remaining cells were mouse cell-depleted and used for additional surface- or intracellular human antigen staining. For mouse cell depletion, BM cells flushed from the femurs and tibia of mice were processed with the Mouse Cell depletion Kit (Miltenyi Biotec) according to the manufacturer’s instructions. Thymus was smashed and resuspended in PBS 2% BSA, and spleen was smashed, lysate with ACK Lysing Buffer (ThermoFisher) and resuspended in PBS 2% BSA. After processing, all samples were stained for surface marker and analyzed by flow cytometry. In some experiments, secondary transplantations were performed upon intravenous injection of 10^6^ human CD34+ harvested and purified (CD34 MicroBead Kit – following manufacturer instruction) from the BM of primary engrafted NSGW41 mice to NSG mice (16 weeks).

#### Mobilization *in vivo*

NSGW41 mice were injected i.v. with CD34^+^ cells as previously described above. After stable engraftment (10 weeks after injection), mice were treated for mobilization. G-CSF (Lenograstim, 250μg/kg/day) was delivered for 7 days through osmotic pumps positioned subcutaneously (s.c.) (Micro-osmotic pump 1007D, Alzet). At days 6 and 7 after pumps implantation, mice received i.p. injections of AMD3100 (Mozobil, 5mg/kg/day) and BIO5192 (R&D System, 1mg/kg/day). 6 hours after last i.p. injections, mice were transplanted i.v. with mobilized-derived CD34^+^ cells, from the same donor, transduced with LV-GFP vector for stable overexpression and electroporated with mRNA for transient overexpression of the indicated gene products.

Different protocols were tested in Cd40lg^-/-^ mice: (i) s.c. pump delivering 250μg/kg/day of G-CSF for 7 days (G7); (ii) s.c. pump delivering 250μg/kg/day of G-CSF for 7 days, with i.p. injections of AMD3100 (5mg/kg/day) on day 6 and 7 (G7A); (iii) s.c. pump delivering 250μg/kg/day of G-CSF for 7 days, with i.p. injections of AMD3100 (5mg/kg/day) and BIO5192 (1mg/kg/day) on day 6 and 7 (G7AB); (iv) s.c. pump delivering 125μg/kg/day of G-CSF for 7 days, with i.p. injections of AMD3100 (5mg/kg/day) and BIO5192 (1mg/kg/day) on day 6 and 7 (G7AB-H); (v) G-CSF delivered by i.p. injections (125 μg/kg) every 12 hours for four days, with AMD3100 s.c. injections (5 mg/kg) 14 hours after the last dose of G-CSF (G5A); (vi) G-CSF delivered by i.p. injections (125 μg/kg) every 12 hours for four days, with AMD3100 (5 mg/kg) and BIO5192 (1mg/kg) s.c. injections 14 hours after the last dose of G-CSF (G5AB); (vii) s.c. pump delivering 250μg/kg/day of G-CSF for 3 days, with i.p. injections of AMD3100 (5mg/kg/day) and BIO5192 (1mg/kg/day) on day 6 and 7 (G3AB); (viii) s.c. pump delivering 125μg/kg/day of G-CSF for 3 days, with i.p. injections of AMD3100 (5mg/kg/day) and BIO5192 (1mg/kg/day) on day 6 and 7 (G3AB-H); and (ix) AMD3100 (5mg/kg/day) and BIO5192 (1mg/kg/day) i.p. injected for three days.

Bone marrow vacancy and estimated chimerism were calculated based on the following formulas in Cd40lg^-/-^ mice (Table 1):

To determine the total number of SLAM HSC per mouse at steady state (Figure 2G, left panel), lower limbs (which account for 20% of the total BM, Nombela-Arrieta and Manz, 2017) were collected in a define volume and counted, paralleled with their characterization through FACS. We found 2500 SLAM HSC/lower limbs, therefore reaching a total of 12 500 SLAM HSC/mouse (2500^∗^100/20). This number is in accordance with other published paper (Chen et al., 2008; Karpova et al., 2017; Singh et al., 2020). To determine the total number of SLAM HSC that egressed from the BM post-G7AB mobilization, we examined the BM of mobilized mice. 1100 SLAM HSC were recovered in the lower limbs, reaching a total of 5500 SLAM HSC in the BM of mobilized mice. Therefore, to determine the number of SLAM HSC egressed, the number of SLAM HSC present post-mobilization was subtracted from the total number of SLAM HSC present in the steady state mouse, leading to an assessment of 7000 SLAM HSC egressing from the BM (12500 – 5500).

The mobilized SLAM HSC / mL was calculated based on the bleeding performed at the peak of mobilization in mobilized mice (Figure 2G, right panel). The blood was analyzed through hematocytometer (WBC/mL) and FACS staining (% subpopulation). Based on the WBC and the percentage of each subpopulation, the mobilized SLAM HSC / mL was estimated. To calculate the total number of mobilized SLAM HSC, the concentration of mobilized SLAM HSC/mL was multiplied by the total blood volume estimated to 1.5mL. Thereby, counts of SLAM HSC in the circulation of mobilized mice (Figure 2G, right panel) were valued to 3500 SLAM HSC/mL, leading to an estimation of 5250 total SLAM HSC total in the circulation (2500^∗^1.5), corresponding to 75% of the 7000 SLAM HSC egressed from the BM.

**Table:** 

Figure 2G		Steady state mice (Sham)	Mobilized mice (G7AB)
Left panel	# SLAM HSC in lower limbs	2500	1100
Left panel	# SLAM HSC total BM ^Lower limbs = 20% total BM^	12500	5500
Left panel	#SLAM HSC egressed (# SLAM HSC total BM Sham - # SLAM HSC total BM mobilized)	N/A	12500 – 5500 = 7000
Right panel	# SLAM HSC circulation (Total blood volume = 1.5mL)	28^∗^1.5 = 43	3500^∗^1.5 = 5250
	% SLAM HSC in circulation to SLAM HSC egressed from the BM	N/A	5250^∗^100/7000 = 75%

##### Concerning the transplanted Lin^-^ BM cells

2x10^6^ Lin^-^ BM cells were used / transplantation. Upon purification of Lin^-^ cells from the BM, we characterized the Lin^-^ population using FACS and determined the percentage of LSK (average 9% Lin-; 9x10^4^ LSK/million of Lin^-^) and SLAM HSC (average 2%; 2000 SLAM HSC/million of Lin^-^) subpopulation. We next calculated the transplanted number of LSK and SLAM HSC from 2x10^6^ Lin^-^ cells, corresponding to 4000 SLAM HSC total.

The ratio recipient to donor was determined by dividing the mobilized recipient SLAM HSC by the transplanted SLAM HSC. As 2x10^6^ Lin^-^ were transplanted each time, except for the dose response experiment (Figure S2M), the SLAM HSC transplanted always correspond to 4000 (Table 1).

$$BM\;vacancy\left(\%\right)=\frac{Total\;mobilized\;SLAM\;HSC*100}{Total\;SLAM\;HSC\;in\;the\;BM\;}$$

$$Estimated\;chimerism\left(\%\right)=\frac{BM\;vacancy}{\left(\frac{total\;SLAM\;HSC\;mobilized}{total\;SLAM\;HSC\;infused}\right)+1}$$

Humanized bone marrow vacancy and estimated chimerism were calculated based on the following formulas:

$$BM\;vacancy\left(\%\right)=\frac{Total\;mobilized\;CD34*100}{Total\;CD34\;in\;the\;BM\;}$$

$$Estimated\;chimerism\left(\%\right)=\frac{BM\;vacancy}{\left(\frac{total\;CD34\;mobilized}{total\;CD34\;infused}\right)+1}$$

#### Molecular analysis

For HDR digital droplet PCR (ddPCR) analysis, 5–50 ng of gDNA were analyzed using the QX200 Droplet Digital PCR System (Bio-Rad) according to the manufacturer’s instructions. HDR ddPCR primers and probes were designed on the junction between the vector sequence and the targeted locus. Human TTC5 (Bio-Rad) was used for normalization. DNA was extracted using Qiamp DNA micro kit (Qiagen) or Qiamp DNA mini kit according to starting number of cells (as suggested by manufacturers). DNA was subsequently quantified and checked for purity. Vector copies per diploid genome (vector copy number, VCN) were quantified by ddPCR starting from 5-50 ng of template gDNA using the following primers (HIV sense: 5′-TACTGACGCTCTCGCACC-3′; HIV antisense: 5′-TCTCGACGCAGGACTCG-3′) and probe (FAM-ATCTCTCTCCTTCTAGCCTC-MGBNFQ) against the primer binding site region of LVs. Endogenous DNA amount was quantified by a primer/probe set against the human telomerase gene (Telo sense: 5′- GGCACACGTGGCTTTTCG-3′; Telo antisense: 5′-GGTGAACCTCGTAAGTTTATGCAA-3′; Telo probe: VIC 5′-TCAGGACGTCGAGTGGACACGGTG-3′ TAMRA). Copies per genome were calculated by the formula = [ng LV/ng endogenous DNA] × [no of LV integrations in the standard curve]. All reactions were carried out in duplicate. Each ddPCR run carries an internal control in the form of a CEMA301 cell line stably carrying a single vector integrant previously validated by Southern blot analysis.

For gene expression analyses, total RNA was extracted using RNeasy Plus Micro Kit (QIAGEN), according to the manufacturer’s instructions and DNAse treatment was performed using RNase-free DNAse Set (QIAGEN). Complementary DNA was synthesized with SuperScript VILO IV cDNA Synthesis Kit (Thermo Fisher) with EzDNAse treatment. cDNA was then used for quantitative PCR (qPCR) in a Viia7 Real-time PCR thermal cycler using TaqMan Gene Expression Assays (Applied Biosystems) mapping to human IRF7, OAS1, ISG15 and RIG-I genes. Data were analyzed with QuantStudio Real-Time PCR software v.1.1 (Applied Biosystem). Relative expression of each target gene was first normalized to HPRT and then represented as fold changes (2-ΔΔCt) relative to the untreated cells.

### Quantification and statistical analysis

Here, the n indicates the number of biologically independent samples, animals, or experiments. For some experiments, different HSPC donors were pooled. Data were summarized as mean ± SEM. Inferential techniques were carried out whenever they were necessary for the interpretation of the data, otherwise descriptive statistics are reported. The Mann–Whitney test was performed to compare two independent groups, while in presence of more than two independent groups the Kruskal–Wallis test followed by post hoc analysis using Dunn’s test was used. In presence of dependent observations and longitudinal comparisons the mixed-effects model (restricted maximum likelihood, REML) were performed, followed by post hoc analysis with Sidak’s test (when groups =2) or Tukey’s test (when groups >2) and/or by post hoc analysis with Dunnett’s test (within group). In all the analyses, P-values less than 0.05 were considered significant (^∗^P < 0.05, ^∗∗^P < 0.01, ^∗∗∗^P < 0.001, ^∗∗∗∗^P < 0.0001. “ns” means non-significance). All statistical analyses were performed using R 3.5.0 (http://www.R-project.org/) or GraphPad Prism v8.

Following details of the statistical analysis performed for each panel:

Figure 1 *. Long-term donor chimerism is established by mobilization-based HSCT (M-HSCT)*

(B) Kruskal–Wallis test, followed by post hoc analysis with Dunn’s test. (D) Longitudinal comparisons, performed by mixed-effects model (REML), followed by post hoc analysis with Tukey’s test (between groups) or by post hoc analysis with Dunnett’s test (within groups). (E) Comparison of lineages between CD45.1 and CD45.2 cells performed at the last time point by mixed-effects model (REML), followed by post hoc analysis with Dunnett’s. (F-I) Mixed-effects model (REML), followed by post hoc analysis with Tukey’s test (between groups) or by post hoc analysis with Dunnett’s test (within groups). (J-K) Comparison of lineages between CD45.1 and CD45.2 cells, performed by mixed-effects model (REML), followed by post hoc analysis with Dunnett’s test.

Figure 2 *. M-HSCT allows establishing sufficient donor chimerism to rescue the HIGM1 phenotype*

(B-D) Kruskal–Wallis test performed for the 3-hour timepoint, followed by post hoc analysis with Dunn’s test. (G) Mann–Whitney test performed. (H) Longitudinal comparisons, performed by mixed-effects model (REML), followed by post hoc analysis with Sidak’s test or by post hoc analysis with Dunnett’s test (within groups). (I-K) Comparison of lineages between WT and *Cd40lg*^*-/-*^ cells performed by mixed-effects model (REML), followed by post hoc analysis with Dunnett’s. (L) Mixed-effects model (REML), followed by post hoc analysis with Tukey’s test. (M) Kruskal–Wallis test, followed by post hoc analysis with Dunn’s test.

Figure 3 *. M-HSCT allows efficient donor to recipient exchange of HSPCs within the human niche of hematochimeric mice*

(B) Kruskal–Wallis test, followed by post hoc analysis with Dunn’s test. (C) Mann–Whitney test performed. (G-H) Mann–Whitney test performed. (I) Mixed-effects model (REML), followed by post hoc analysis with Sidak’s test (between groups) or by post hoc analysis with Dunnett’s test (within groups). (J) Comparison of lineages between human CD45^+^ and GFP^+^ cells performed at the last time point by mixed-effects model (REML), followed by post hoc analysis with Dunnett’s test (within groups). (K-L) Mixed-effects model (REML), followed by post hoc analysis with Sidak’s test. (M) Comparison of lineages between human CD45^+^ and CD45^+^/GFP^+^ cells by mixed-effects model (REML), followed by post hoc analysis with Dunnett’s test (within groups). (N). Mixed-effects model (REML), followed by post hoc analysis with Sidak’s test (between groups) or by post hoc analysis with Dunnett’s test (within groups). (O) Kruskal–Wallis test was performed, followed by post hoc analysis with Dunn’s test. (P) Mann–Whitney test performed. (Q) Comparison of lineages between human CD45^+^ and CD45^+^/GFP^+^ cells performed by mixed-effects model (REML), followed by post hoc analysis with Dunnett’s test (within groups). (R) Mixed-effects model (REML), followed by post hoc analysis with Sidak’s test (between groups) or by post hoc analysis with Dunnett’s test (within groups). (S) Kruskal–Wallis test was performed, followed by post hoc analysis with Dunn’s test. (T) Mann–Whitney test performed.

Figure 4 *. Transient overexpression of CXCR4 increases chimerism in the humanized context, following M-HSCT*

(A-D) Mixed-effects model (REML), followed by post hoc analysis with Sidak’s test. (E-F) Kruskal–Wallis test was performed, followed by post hoc analysis with Dunn’s test. (I) Kruskal–Wallis test was performed, followed by post hoc analysis with Dunn’s test. (J) Longitudinal comparisons, performed by mixed-effects model (REML), followed by post hoc analysis with Sidak’s test. (K) Comparison of lineages between human CD45^+^ and GFP^+^ cells performed at the last time point by mixed-effects model (REML), followed by post hoc analysis with Dunnett’s test (within groups). (L) Comparison of lineages between human CD45^+^cells by mixed-effects model (REML), followed by post hoc analysis with Dunnett’s test (within groups). (M) Mixed-effects model (REML), followed by post hoc analysis with Sidak’s test. (O-P) Two-way ANOVA followed by post hoc analysis with Tukey’s test. (Q) Two-way ANOVA followed by post hoc analysis with Tukey’s test. (R) Comparison of lineages between human CD45^+^ and CD45^+^/GFP^+^ cells performed by mixed-effects model (REML), followed by post hoc analysis with Dunnett’s test (within groups). (S) Mixed-effects model (REML), followed by post hoc analysis with Tukey’s test (between groups) or by post hoc analysis with Dunnett’s test (within groups). (T) Two-way ANOVA followed by post hoc analysis with Tukey’s test. (U) Two-way ANOVA followed by post hoc analysis with Tukey’s test.

Figure 5 *. M-HSCT confers significant advantage to gene edited cells when paired with an engraftment enhancer*

(A-B) Kruskal–Wallis test, followed by post hoc analysis with Dunn’s test. (C-D) Mixed-effects model (REML), followed by post hoc analysis with Tukey’s test. (E-G) Kruskal–Wallis test, followed by post hoc analysis with Dunn’s test. (H) Kruskal–Wallis test, followed by post hoc analysis with Dunn’s test.
